# Diagnostic Modalities of Non-Alcoholic Fatty Liver Disease: From Biochemical Biomarkers to Multi-Omics Non-Invasive Approaches

**DOI:** 10.3390/diagnostics12020407

**Published:** 2022-02-04

**Authors:** Eirini Martinou, Marinos Pericleous, Irena Stefanova, Vasha Kaur, Angeliki M. Angelidi

**Affiliations:** 1 Hepatobiliary and Pancreatic Surgery Department, Royal Surrey County Hospital, Guildford GU2 7XX, UK; 2 Faculty of Health and Medical Sciences, University of Surrey, Guildford GU2 7XH, UK; mpericleous@nhs.net; 3 Department of Gastroenterology and Hepatology, Royal Surrey County Hospital, Guildford GU2 7XX, UK; 4 Department of General Surgery, Frimley Health NHS Foundation Trust, Camberley GU16 7UJ, UK; irena.stefanova@nhs.net; 5 Department of Upper Gastrointestinal and Bariatric Surgery, St George’s Hospital, London SW17 0QT, UK; vasha.kaur@nhs.net; 6 Department of Medicine, Beth Israel Deaconess Medical Center, Harvard Medical School, Boston, MA 02215, USA

**Keywords:** non-alcoholic fatty liver disease, biomarkers, diagnostic, omics

## Abstract

Non-Alcoholic Fatty Liver Disease (NAFLD) is currently the most common cause of chronic liver disease worldwide, and its prevalence is increasing globally. NAFLD is a multifaceted disorder, and its spectrum includes steatosis to steatohepatitis, which may evolve to advanced fibrosis and cirrhosis. In addition, the presence of NAFLD is independently associated with a higher cardiometabolic risk and increased mortality rates. Considering that the vast majority of individuals with NAFLD are mainly asymptomatic, early diagnosis of non-alcoholic steatohepatitis (NASH) and accurate staging of fibrosis risk is crucial for better stratification, monitoring and targeted management of patients at risk. To date, liver biopsy remains the gold standard procedure for the diagnosis of NASH and staging of NAFLD. However, due to its invasive nature, research on non-invasive tests is rapidly increasing with significant advances having been achieved during the last decades in the diagnostic field. New promising non-invasive biomarkers and techniques have been developed, evaluated and assessed, including biochemical markers, imaging modalities and the most recent multi-omics approaches. Our article provides a comprehensive review of the currently available and emerging non-invasive diagnostic tools used in assessing NAFLD, also highlighting the importance of accurate and validated diagnostic tools.

## 1. Introduction

Non-Alcoholic Fatty Liver Disease (NAFLD) is the most common cause of chronic liver disease in the developed world [1]. The American Association for the Study of Liver Diseases (AASLD) defines NAFLD as excessive hepatic fat accumulation with evidence of hepatic steatosis either on histology or radiological imaging; no significant alcohol consumption; lack of competing causes for hepatic steatosis and no concurrent causes of chronic liver disease [1,2]. Two distinctive histological forms of NAFLD are associated with different prognoses: non-alcoholic fatty liver (NAFL) and non-alcoholic steatohepatitis (NASH). The diagnosis of NAFLD requires the presence of >5% HS without evidence of hepatocellular injury, whilst NASH is defined by >5% steatosis with inflammation and hepatocellular injury [2]. NASH includes a different severity spectrum of liver disease, ranging from fibrosis, cirrhosis to hepatocellular carcinoma (HCC) [3]. NAFLD is present in approximately 23.5% of the adult US population and 17 to 46% of adults in western countries [3,4]. The high prevalence of NAFLD in the industrialised nations is associated with the observed increased rates of obesity and metabolic syndrome (hypertension, type 2 diabetes (T2DM) and dyslipidaemia). A meta-analysis, including 8.5 million people from 22 countries, reported that 80% of patients with NASH are either overweight or obese, 72% suffer from dyslipidaemia and 44% have a diagnosis of T2DM. Moreover, “lean” NAFLD, affecting normal-weight individuals, is reported in 7% of Americans [5,6].

The pathophysiology of NAFLD is complex and multifactorial. In recent years, there has been a shift away from the two-hit hypothesis introduced in the 90s. This theory involved a first hit in the form of insulin resistance (IR), followed by a second hit characterised by oxidative stress with subsequent lipid peroxidation, the release of inflammatory cytokines and adipokines and mitochondrial dysfunction, leading to the development of NASH [7]. It is now evident that the underlying mechanism of NAFLD is more intricate, and it constitutes multiple parallel factors acting synergistically in a genetically predisposed individual as part of an interorgan cross-talk between adipose tissue, liver, pancreas and the gut [3,8]. IR resulting from an unhealthy diet and sedentary lifestyle has a key role in inducing hepatic de novo lipogenesis, with a subsequent influx of free fatty acids (FFAs) into hepatocytes, as well as promoting adipose tissue dysfunction with a release of adipokines and inflammatory cytokines. Fat deposition in the liver leads to increased lipotoxicity, followed by mitochondrial dysfunction and oxidative stress [9,10,11]. At the same time, altered gut microbiome leads to increased bowel permeability and absorption of FFAs, raising their circulation levels and stimulating proinflammatory cytokine production. With the background of genetic factors and epigenetic changes, these events might affect hepatocyte fat content and liver inflammatory environment, causing a state of chronic inflammation with or without progression to hepatocyte death, activation of hepatic stellate cells and deposition of a fibrous matrix [8].

Considering the above-described mechanisms, NAFLD is now perceived as a hepatic manifestation of a systemic metabolic disorder [12]. Eslam et al. have proposed a new nomenclature for NAFLD—metabolic associated fatty liver disease (MAFLD), which is defined by its own set of positive criteria rather than exclusion criteria. This allows MAFLD diagnosis to be made regardless of alcohol consumption and in the presence of other chronic liver diseases [13,14]. NAFLD manifests with hepatic and extra-hepatic complications. The natural progression of NAFLD to cirrhosis is slow, but fibrosis rapidly progresses in 20% of the cases. The reported rate of progression is one fibrosis stage every 14 years in NAFLD and every 7 years in NASH, which is doubled in arterial hypertension [15]. The global prevalence of NAFLD is estimated at 24% [5], whilst NASH affects approximately 1.5% to 6.5% of adults worldwide. A quarter of patients with NASH already have F2 fibrosis at the time of diagnosis [5]. One-third of patients with advanced fibrosis develop cirrhosis, with 5–10% suffering a decompensation [16]. Furthermore, 1–2% of patients with NASH cirrhosis per year develop HCC [17]. NAFLD-related cirrhosis and HCC have become one of the top three leading indications for liver transplantation in the US [4,17,18]. The most common cause of death in NAFLD patients is cardiovascular disease (CVD), whilst liver-related mortality takes third place after cancer [19,20]. Patients with NAFLD may also exhibit ectopic fat deposition in other organs, apart from the liver [21,22]. These abnormal intra- or perio-organ fat deposits (such as epicardial, intramuscular, perivascular, perirenal and peripancreatic intramuscular adiposity) have been found to be associated with increased cardiovascular risk [23]. Therefore, this ectopic fat may also contribute to the high cardiovascular risk observed in NAFLD patients [21,22]. Additionally, there are various NAFLD-related extrahepatic complications, including chronic kidney disease and T2DM, which are also associated with severity of NAFLD, progression to NASH, development of fibrosis and HCC [24,25].

The growing clinical consequences of NAFLD are leading to an increased economic burden worldwide. A model estimated that annual medical costs associated with all NAFLD patients are USD 103 billion in the USA and EUR 35 billion in four European countries (UK, France, Italy and Germany) [4]. As NAFLD prevalence is parallel to the increasing rates of obesity, the expected economic burden could increase to USD 1003 trillion in the USA and EUR 334 billion in Europe within 10 years [4]. This raises the question of whether screening for NAFLD could be justified. Currently, all society guidelines recommend against systematic screening for NAFLD in the general population. This is a result of insufficient evidence of long-term benefits of screening, uncertain accuracy of non-invasive diagnostic tests, lack of effective treatment for NASH and cost-effectiveness analysis [1,4].

However, screening in high-risk groups, such as patients with metabolic disorders (T2DM, obesity, hypertension, dyslipidaemia), is still open for debate. Considerable variability in society recommendations still exists. AASLD advises against screening even in populations at risk but recommends a “high index of suspicion” in diabetes [1]. National Institute of Health and Care Excellence (NICE) guidelines have similar approach to the AASLD; however, their high index of suspicion includes patients with T2DM and metabolic syndrome [26]. Meanwhile, the European guidelines state that all patients with obesity and metabolic syndrome should be screened due to the prognostic implications of progressive disease [3]. The Asian guidelines consider screening for high-risk groups, such as patients with diabetes and obesity, which does not account for the higher prevalence of “lean” NAFLD in Asian countries [27]. Early recognition and intervention are vital to improve clinical outcomes and reduce the economic and health burden of NAFLD.

For the diagnosis of NAFLD, the ultrasound remains the first-line modality, despite being less reliable if HS is <20% [28]. Other tools, such as magnetic resonance imaging, have high sensitivity but are unavailable for wide use. Vibration-controlled transient elastography (FibroScan system) is a non-invasive test that allows for grading of fibrosis stage based on liver stiffness. However, none of these modalities can definitively differentiate simple steatosis from steatohepatitis, hence liver biopsy remains the gold standard. Due to its high cost and invasive nature, liver biopsy is reserved only for selected patients [1]. There is a recognised ongoing need for a non-invasive tool that could accurately identify NASH and also discern low- and high-risk individuals for advanced fibrosis, as high-risk patients would need closer surveillance and management [1].

The present study aims to provide a comprehensive review of the non-invasive diagnostic tools used in the assessment of NAFLD, ranging from biochemical markers and imaging modalities to multi-omics approaches. Therefore, a systematic literature review, following the Preferred Reporting Items for Systematic reviews and Meta-Analyses (PRISMA) guidelines, was not performed. We searched for relevant reviews and original articles using Medline, PubMed, Web of Science and Google Scholar databases using search terms related to the disease and diagnostic modalities discussed in the current review without publication time restrictions. In the present review article, current and evolving biochemical and imaging diagnostic tools are presented in detail. Moreover, special emphasis is given to the emerging role of omics in the last section of this review article.

## 2. Biochemical Diagnostic Markers

As proposed by the clinical care pathway from the American Gastroenterology Association (AGA), the first biochemical evidence alluding to a diagnosis of Non-Alcoholic Fatty Liver Disease (NAFLD) is the abnormal serum liver biochemistry [29].

Even though the majority of patients with NAFLD and NASH are asymptomatic, a mild elevation of liver function tests (usually less than five times the upper limit of normal) might be observed [29]. It is important to highlight that the degree of aminotransferase rise does not reflect the degree of hepatocellular injury associated with NAFLD/NASH [30]. AST/ALT ratio (AAR) is a fundamental index used for the non-invasive staging of liver fibrosis, and it is included in the BARD score, NAFLD fibrosis score and FIB-4 score.

γ-glutamyltransferase (GGT) can be frequently elevated in patients with NAFLD, and it is associated with advanced fibrosis and increased mortality in these patients [31]. GGT value is a component of various liver fibrosis diagnostic models, including FibroTest and Hepascore. The Prothrombin/International Normalised Ratio (INR) and albumin are also markers of hepatic synthetic function. Low platelets in liver disease are often used as a surrogate marker for splenomegaly and portal hypertension, but they can also be suppressed in situations of increased bone marrow burden, including alcohol excess, iron overload, drugs and viridae [32]. Increased destruction of platelets is not unusual in hepatology patients and can result from shear stress, fibrinolysis and bacterial translocation [32]. The platelet count is therefore used in various non-invasive tests for diagnosis of significant hepatic fibrosis, including FIB-4, NAFLD Fibrosis Score (NAFLD FS), Fib-4 and APRI score.

**Figure 1:**
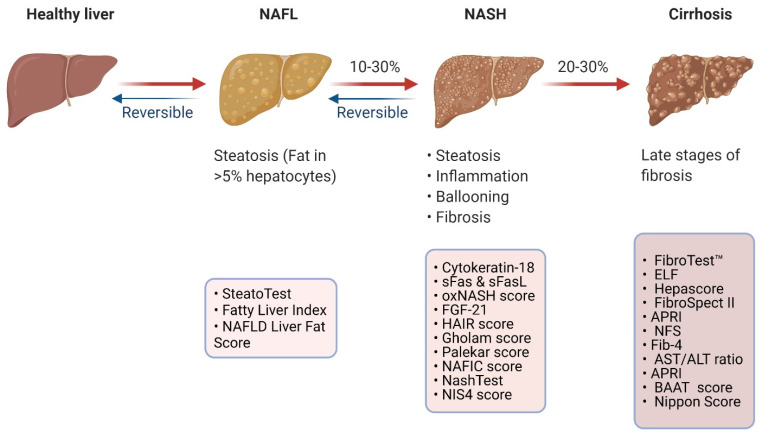
Overview of biochemical indices and diagnostic models discussed in the paper (created with BioRender.com, accessed on 21 December 2021). APRI: AST/PLT ratio index, ALT: Alanine aminotransferase, AST: Aspartate aminotransferase: BAAT: Body mass index, age, ALT and triglycerides, ELF: European Liver Fibrosis panel, FGF-21: Fibroblast growth factor 21, HAIR: Hypertension, ALT and insulin resistance NAFL: Non-alcoholic fatty liver, NAFLD: Non-alcoholic fatty liver disease, NASH: Non-alcoholic steatohepatitis, NAFIC: Ferritin, insulin and collagen NFS: NAFLD Liver Fat Score, sFas: Soluble Fas, sFasL: Fas ligand.

The high volumes of patients with NAFLD have necessitated the consideration of a traffic light system and flow-charts to prognosticate clinical outcomes and fashion care plans for patients [33,34]. Multiple societies have considered this risk-stratifying approach for patients with NAFLD/NASH in their guidance and endorse the use of routine liver function tests coupled with serum fibrosis markers (e.g., FIB-4, NAFLD Fibrosis Score, FibroTest, FibroMeter, ELF), to dictate patient referral thresholds and follow-up plans [2,3,35]. In this section, biochemical diagnostic biomarkers predicting the presence of NAFLD, NASH and advanced fibrosis are discussed and summarised in Figure 1.

### 2.1. Biomarkers Predicting the Presence of NAFLD

In addition to the biochemical markers discussed above, more complex diagnostic models have been suggested to predict the presence of hepatic steatosis in the general population. By way of illustration, SteatoTest, Fatty Liver Index and NAFLD Liver Fat Score are presented.

#### 2.1.1. SteatoTest

SteatoTest aims to estimate the degree of steatosis in patients by using the six parameters of the Fibrotest adjusted for age, gender and Body Mass Index (BMI), plus serum lipids (cholesterol and triglycerides) and glucose [36,37]. The authors analysed the results of 884 patients and validated the outcomes in three validation cohorts. Validation group 1 contained untreated hepatitis C (HCV) patients; validation group 2 included patients with hepatitis C virus (HCV) infection with sustained virological response; and validation group 3 included patients with Alcoholic Liver Disease (ALD). SteatoTest provides scores in the range of zero to one, with higher scores indicating a higher degree of steatosis. The Area Under the Receiver Operating Characteristic curve (AUROC) for the test was 0.79. Using a score of 0.30, the test was found to have a sensitivity of 91% and specificity of 70% for the diagnosis of Grade 2–4 steatosis [36]. The test has 63% and 93% Positive Predictive Value (PPV) and Negative Predicative Value (NPV), respectively [36].

#### 2.1.2. Fatty Liver Index (FLI)

FLI has been developed by Bedogni et al., who considered 13 variables in approximately 500 patients [38]. The model identified waist circumference, BMI, triglycerides and GGT as predictors of hepatic steatosis. The model is scored from 0–100. Scores of <30 were predictive of a low likelihood of hepatic steatosis, whilst a score of >60 ruled in fatty liver disease. The sensitivity, specificity, positive likelihood radio and negative likelihood ratio were 87%, 86%, 4.3 and 0.2, respectively [38]. The AUROC was 0.85 (95% CI, 0.82–0.89). The model has also been validated by other authors [39,40].

#### 2.1.3. NAFLD Liver Fat Score (NFS)

Kotronen et al. used a combination of metabolic and genetic parameters to develop a model for predicting NAFLD using data from nearly 500 patients [41]. The score uses five parameters, i.e., metabolic syndrome, T2DM, fasting insulin, AST and the AST/ALT ratio. With a cut-off score of −0.640, the model was found to have sensitivity and specificity of 86% and 71%, respectively. The test has an AUROC of 0.88 (95% CI, 0.84–0.92). In the same study, the authors also developed a model to predict the fat percentage [41].

### 2.2. Biomarkers Predicting the Presence of NASH

Whilst the detection of simple steatosis in NAFLD might be feasible on imaging, the biopsy is considered the optimal method of accurate assessment of NAFLD staging [42]. However, due to its invasive nature and the potential associated complications and limitations, it tends to be reserved for specific groups of patients [42]. Therefore, measurement of individual blood markers (or panels made up of these markers) may allude to a diagnosis of NASH and provide a non-invasive approach in distinguishing NASH from NAFLD.

#### 2.2.1. Biomarkers of Apoptosis

Cytokeratin (CK-18), or KRT18, is a marker of apoptosis and probably the most widely studied biomarker for predicting the presence of non-alcoholic steatohepatitis (NASH) in patients with hepatic steatosis [43]. During apoptosis, the activation of caspases leads to the cleavage of the CK-18, which is a filament protein in the hepatocytes. It provides a direct measurement of hepatocellular damage and apoptosis [43]. In two meta-analyses, CK-18 had a pooled AUROC to detect NASH of 0.82 (95% CI, 0.76–0.88) with a sensitivity of 66–78% and specificity of 82–87% [44,45]. The heterogeneously reported diagnostic accuracy of CK-18 has made the choice of a reliable cut-off for the test very difficult. Moreover, the lack of commercially available CK-18 tests and their reduced sensitivity resulted in its limited clinical use. Tamimi et al. combined CK-18 with soluble Fas (sFas) to improve diagnostic accuracy and calculated AUROC of 0.93 (95% CI, 0.88–0.98), with a sensitivity of 88% and a specificity of 89% [46]. Huang et al. combined CK-18 with uric acid and reported sensitivity and specificity of 60.9% and 64.2% for F1 fibrosis, 96.4% and 28.6% for F2 fibrosis, and 97.1% and 54.1% for F3–4 fibrosis [47]. Younossi et al. combined CK-18 with serum Adiponectin and serum Resistin, and reported AUROC of 0.908 (95% CI, 0.814–0.964), sensitivity of 95.45% and specificity of 70.21% for differentiating NASH from fatty liver disease [48]. Anty et al. combined CK-18 with ALT and metabolic syndrome (the Nice Model) and reported AUROC of 0.88, sensitivity of 0.84 and specificity of 0.86 [49].

Soluble Fas (sFas) and Fas ligand (sFasL) are markers of the extrinsic pathway of hepatocyte apoptosis. High levels have been associated with NASH [50]. Alkhouri et al. have demonstrated that sFasL has an AUROC of 0.714 (95% CI, 0.618–0.810) and can be combined in NASH panels, such as the NASH apoptosis score [50].

#### 2.2.2. Biomarkers of Inflammation

Inflammation is one of the hallmarks of NASH. Several inflammatory markers have been considered as circulating biomarkers for the diagnosis of NASH and fibrosis, including C-reactive protein (CRP), tumour necrosis factor (TNF), interleukin-8 (IL-8) and C-X-C Motif chemokine ligand 10 (CXCL10). Increased levels of total plasminogen activator inhibitor, activated plasminogen activator inhibitor 1 (aPAI1), IL-8 and soluble interleukin-1 (IL-1) receptor 1 were found to be raised in NASH, but only aPAI1 has been found to be significantly associated with a diagnosis of NASH in a recent study by Ajmera et al. [51]. Serum TNF-a is another marker of inflammation that has been shown to correlate with the presence of NAFLD and NASH [52]. It has been suggested that TNF-a might be involved in the development of insulin resistance, inflammation and fibrosis in patients with NASH, therefore, anti-TNF medication may present a potential therapeutic target in patients with NASH [52,53,54]. The role of interleukin-6 (IL-6) concerning the severity of hepatocyte inflammation, staging of fibrosis and insulin resistance in NASH and NAFLD, has been reviewed in the literature by various authors 2324. IL-8 has been shown to be elevated in patients with NAFLD and to have a strong correlation with NASH [52,55].

#### 2.2.3. Biomarkers of Lipid Oxidation

Oxidative stress can contribute to hepatocyte damage and may encourage the development of NASH from NAFLD. Measurement of lipid oxidation products requires mass spectrometry, which largely limits their use in clinical practice. Oxidation products 9-Hydroxyoctadecadienoic acid (9-HODE), 13- Hydroxyoctadecadienoic acid (13-HODE), 9-oxooctadecadienoic acid (9-oxoODE) and 13-oxooctadecadienoic acid (13-oxoODE) were found to be significantly elevated in NASH, but not in NAFLD patients [56].

#### 2.2.4. Adipokines and Hormones

There are several adipocytokines that have been implicated in the pathogenesis of NASH [57]. Low levels of Adiponectin have been associated with metabolic syndrome, NAFLD and NASH. Adiponectin may be able to predict the clinical course of NAFLD even before patients present with elevated inflammatory markers [58,59]. Visfatin is another important adipocytokine. Low Visfatin levels in the serum and visceral adipose tissue have been correlated with NAFLD, NASH, cirrhosis, metabolic syndrome and insulin resistance [52,60,61]. Fibroblast growth factor 21(FGF-21) is released in the circulation because of liver cell apoptosis, inflammation, fat metabolism and oxidative stress. FGF-21 levels have been associated with hepatic steatosis. In a meta-analysis, FGF-21 was found to be a potential biomarker for liver steatosis and its progression to steatohepatitis, but it had low sensitivity and specificity, i.e., 0.62 and 0.78, respectively [62]. FGF-21 analogues have been shown to significantly reduce the amount of hepatic steatosis in NASH patients in clinical trials. Therefore, FGF-21 can be a target therapeutic biomarker for NAFLD/NASH [63].

#### 2.2.5. Mathematical Models Predicting NASH

The oxNASH model was developed by Feldstein et al., who used mass spectrometry to measure circulating lipid oxidation products. These products, coupled with age, BMI and transaminases, were computed to predict the presence of NASH in patients with NAFLD. The combination of 13-hydroxyl-octadecadienoic acid (13-HODE)/linoleic acid ratio, age, BMI and AST provides a model with AUROC of 0.74 (95% CI, 0.6, 0.88), sensitivity of 84% and specificity of 63% [56].

There are other predictive models of NASH that combine the use of clinical and biochemical parameters. The HAIR model (hypertension, increased ALT and raised insulin resistance) was developed using surgical samples from 105 bariatric patients and was found to have AUROC of 0.9, sensitivity of 80% and specificity of 89% [64]. Interestingly, the authors identify alcohol consumption as a factor that reduced the risk of NAFLD in bariatric patients [64].

Gholam et al. considered histological findings, insulin resistance, transaminases and metabolic parameters as predictors of NASH in morbidly obese patients. A simple model using AST and diabetes mellitus was found to have an AUROC of 0.82. A model including ALT and HbA1C was highly predictive of fibrosis and had an AUROC of 0.9 [65].

Palekar et al. combined six parameters to design a model to distinguish steatosis from NASH [66]. These included age ≥50 years, female gender, AST, BMI ≥ 30 mg/kg^2^, AST/ALT ratio >0.8 and hyaluronic acid >55. The AUROC for the composite index of all six parameters was 0.76 (95% CI, 0.65–0.88). The group reported a sensitivity of 74%, and a specificity of 66% if three or more risk factors were present. The composite model was also found to be a discriminant for higher stage fibrosis (F3–4), with hyaluronic acid being the strongest predictor [66].

Type IV collagen 7S has been found to be elevated in patients with NASH and advanced fibrosis [67]. The NAFIC score was developed on a Japanese cohort by Sumida et al., and it combines type IV collagen 7S, ferritin and insulin to differentiate NASH from NAFLD [68]. The score ranges from 0–4, and the authors used cut-off score values of 1 and 2 to provide estimations for the performance and accuracy of the NAFIC score. The model had an AUROC of 0.851. For a cut-off NAFIC score 2 and 1, the sensitivity and specificity of the test were 66%, 91%, 94% and 48%. One of the biggest limitations of the study was the exclusion of a significant proportion of diabetic patients based on their treatment regimen [68].

The NashTest was developed by the same group responsible for the development of the AshTest, which aims to predict the presence of alcoholic steatohepatitis in heavy alcohol drinkers [69]. In their study, Poynard et al. recruited 160 NAFLD patients, whose biochemical and histological parameters were evaluated [70]. The results were validated using a separate validation cohort. The NashTest uses thirteen elements to provide scores that can divide the patients into three groups, i.e., diagnostic of NASH, borderline NASH and no NASH. The thirteen parameters used to develop the test were age, sex, height, weight, serum levels of triglycerides, cholesterol, α2-macroglobulin, apolipoprotein A1, haptoglobin, GGT, aminotransferases ALT, AST and total bilirubin. The AUROC was 0.79 (95% CI, 0.69–0.86). The sensitivity of the NashTest was 33% and specificity was 94% [70].

More recently, Harrison et al. described a proprietary blood panel for identifying NASH in patients with metabolic risk factors (NIS4 score) [71]. The panel includes four biomarkers: miR-34a-5p, alpha-2 macroglobulin, YKL-40 and glycated haemoglobin. The score has an AUROC of 0.80 (95% CI, 0.73–0.85). The authors validated their results in two independent cohorts with reproducible outcomes. With the lower cut-off limit of <0.36, the model had a sensitivity of 82% and specificity of 63% for no significant NASH. An upper cut-off limit of >0.63 provided a sensitivity of 87% and specificity of 50.7% for having significant NASH. With these cut-offs, the model had a PPV and NPV of 79% and 78%, respectively [71].

### 2.3. Biomarkers Predicting the Presence of Advanced Fibrosis

#### 2.3.1. Direct Markers of Fibrosis

Direct markers of fibrosis can be divided into three different groups: (a) Markers of matrix deposition (Procollagen I peptide, Procollagen III peptide, Type I collagen, Type IV collagen, Chondrex YKL-40, Laminin, Hyaluronic acid); (b) Markers of matrix degradation (Matrix Metalloproteinase -2, tissue inhibitor of metalloproteinase TIMP -1, -2); (c) Cytokines/chemokines associated with fibrogenesis (transforming growth factor-beta (TGF-β), transforming growth factor-alpha (TGF-α), platelet-derived growth factor (PDGF)).

Many of these markers have been combined in more extensive panels to improve accuracy in predicting the presence of fibrosis. Despite their promising outcomes, their cost and general availability have limited their use in clinical practice.

Hyaluronic acid (HA) is a significant component of the extracellular matrix (ECM), which forms the fibrous tissue in liver fibrosis. Suzuki et al. studied the correlation of HA with hepatic fibrosis in 79 patients with biopsy-proven fibrosis [72]. The AUROC for F2 fibrosis was 0.87, and it was 0.92 for liver cirrhosis. With an HA level of 46.1 μg/L, the test had a sensitivity and specificity of 85% and 80%, respectively. The corresponding PPV and NPV were 51% and 96% [72]. HA has also been studied in relation to many chronic liver diseases, including ALD, hepatitis B virus (HBV), HCV, NAFLD and Primary Biliary Cholangitis (PBC) [73,74,75,76,77].

Procollagen III amino-terminal peptide (PIIINP) is a product of the synthesis of type III collagen or the breakdown of type III collagen strands. PIIINP levels alone do not appear to have a good diagnostic yield for estimating fibrosis in NAFLD and ALD [78]. Pro-C3 is another marker that has been primarily studied in NAFLD patients [79]. Measurement of the propeptide cleaved off the intact collagen molecule (Pro- C3) is more sensitive in estimating the degree of fibrosis [80].

Tissue inhibitors of metalloproteinases -1,-2 (TIMP-1 -2) regulate matrix metalloproteinases and reflect extracellular matrix (ECM) remodelling. TIMPs have not been extensively studied, except in NAFLD and NASH. TIMP-1 -2 have been shown to have very good accuracy in predicting the presence of NASH in obese patients with an AUROC of 0.97. The sensitivity and specificity of TIMP -1 and TIMP -2 were 96.7%, 100%, 93.3% and 100%, respectively [81].

Laminin is a non-collagenous protein synthesised by stellate cells. Raised levels of Laminin have been found to correlate with liver fibrosis in patients with HCV, ALD, NAFLD and portal hypertension [82,83,84]. Laminin was found to have good diagnostic accuracy in predicting fibrosis in patients with NAFLD, with AUROC of 0.87. At a cut-off level of >282 ng/mL, the sensitivity, specificity, PPV and NPV of the test were 82%, 89%, 82% and 89%, respectively [85].

Chondrex (YKL-40) high expression has been found to be associated with increased likelihood of progression to hepatic fibrosis in patients with NAFLD, and it is one of the components of the NIS4 model [86]. Kumagai et al. reported elevated serum YKL-40 levels in a study involving 134 NAFLD patients [87]. Raised YKL-40 levels were found to correlate with an increased likelihood of advanced fibrosis. YKL-40 was found to have AUROC of 0.76, 70% sensitivity, 77% specificity, 68.3% PPV and 78.2% NPV [87]. Though the exact mechanism of action of YKL-40 is not known, it is believed that it is secreted by activated macrophages and attracts endothelial cells, and it modulates angiogenesis during tissue remodelling and repair of various organs, including the liver [88]. Apart from NAFLD, YKL-40 has been proposed as a non-invasive test for many chronic inflammatory and fibrotic liver diseases with high levels of ALD, HCV, HBV [73,86].

Transforming growth factor (TGF)-alpha, TGFbeta and platelet-derived growth factor (PDGF) have been found to play a role in hepatic fibrinogenesis. These cytokines and chemokines have a role in hepatic fibrogenesis. TGF-alpha levels have been found to correlate with Child–Pugh classification, bilirubin levels and cirrhosis. TGF-beta has been studied primarily in HCV-related fibrosis, and levels of PDGF are elevated in chronic liver disease [89,90,91]. The PRTA score (PDGFRβ, thrombocyte, albumin) has been developed to distinguish NAFLD patients with advanced hepatic fibrosis, demonstrating an AUROC of 0.68 (95% CI, 0.52–0.84) [92].

#### 2.3.2. Proprietary Biomarker Panels for Fibrosis

These tests are usually a direct measure of fibrosis or fibrinolysis and have been validated extensively to differentiate patients with significant fibrosis (F2 to F4) vs. those without significant fibrosis (F0 to F1).

The FibroTest™ (FibroSURE in the US) was developed in 2001 as a predictive test for calculating fibrosis in patients with HCV. The score ranges from zero to one. The test was found to have an AUROC of 0.84 and includes bilirubin, GGT, α2-macroglobulin, apolipoprotein A1 and haptoglobin, corrected for age and gender [93]. The test has also been evaluated for use in patients with chronic hepatitis B (and monitoring treatment with Lamivudine) [94,95] and ALD [96,97]. In 2006, Ratziu et al. studied the effectiveness of FibroTest™ in predicting significant fibrosis (F2–F4) in 267 patients. The AUROC was 0.86 (95% CI, 0.77–0.91) and much higher for F3–F4 fibrosis (0.90). With a test cut-off of 0.30, the test had a sensitivity, specificity, PPV and NPV of 77%, 98%, 73% and 90%, respectively [98]. Since the study by Ratziu et al., six further studies reported on the use of FibroTest™ in patients with NAFLD, and the results of these studies have been pooled and analysed in a recent systematic review/meta-analysis [99]. The test was found to have a low performance level for the detection of any fibrosis, ≥F2 fibrosis or ≥F3 fibrosis (all <0.80). However, the test was found to be performing better in diagnosing F4 cirrhosis (AUROC 0.92). Limitations of the test include its reduced performance in patients with early fibrosis stage [99].

Rosenberg et al. first described the ELF test in 2004, in a study involving 1021 patients [100]. The panel uses hyaluronic acid (HA), an amino-terminal propeptide of type III collagen level (PIIINP), and tissue inhibitor of metalloproteinase 1 (TIMP-1). Initially, the panel also included age, which was later removed. The cohort included patients with all forms of chronic liver disease, including HCV, HBV, ALD, PBC, primary sclerosing cholangitis (PSC), autoimmune hepatitis (AIH), hereditary haemochromatosis, post-liver transplantation and cryptogenic cirrhosis [100]. The AUROC for HCV, NAFLD and ALD were 0.73 (95% CI, 0.697–0.848), 0.87 (95% CI, 0.666–1.000) and 0.94 (95% CI, 0.836–1.000), respectively. With a cut-off score of 0.102, the sensitivity and specificity of ELF were estimated to be 90% and 41%, with a PPV of 35% and an NPV of 92% [100]. More recently, in a systematic review and meta-analysis, the ELF test was found to have a sensitivity of >90% for excluding fibrosis in NAFLD patients [101]. Limitations of the test include its reduced performance in patients with early fibrosis stage, low CD4+ T cell or advanced age.

The Hepascore combines bilirubin, GGT, hyaluronic acid, alpha-2-macroglobulin, age and sex. Although it has been used primarily in non-NAFLD cohorts with liver disease, the literature supports its accuracy in NAFLD as well [102,103,104]. It provides an AUROC of 0.82 in patients with NAFLD and advanced fibrosis [105]. In their study, Boursier et al. examined the diagnostic accuracy of Hepascore in 452 NAFLD patients. The model was found to have sensitivity of 67.4%, specificity of 76.1%, PPV of 63.4% and NPV of 79.2% [105]. Comparing Fibrotest, Fib-4, APRI and BARD models, Hepascore and Fib-4 were found to have the highest specificity and PPV for advanced fibrosis. In the same study, Hepascore was found to have the highest AUROC for F4 fibrosis [106].

The FibroSpect II (Prometheus Corp, Lansdale, PA, USA) is a biochemical panel that has been primarily studied in patients with chronic hepatitis C [73,107]. It combines serum hyaluronic acid, tissue inhibitor of metalloproteinase-1 (TIMP-1) and alpha-2 macroglobulin [105]. With a score cut-off of >0.36, the panel had a PPV and NPV values of 74.3% and 75.8%, respectively, for advanced fibrosis [108]. Additionally, the test was found to have 83% sensitivity, 66% specificity and an AUROC of 0.83 [108].

#### 2.3.3. Non-Proprietary Biomarkers and Panels for Fibrosis

Most non-proprietary biomarkers are less expensive and easily accessible to clinicians. They are generally not designed to measure fibrogenesis or fibrinolysis. They capture surrogate processes that have been associated with fibrosis risk factors.

Angelo et al., in a multicentre international study, including 733 patients with biopsy-confirmed NAFLD, created the NAFLD fibrosis score (NFS) intending to identify the presence of F3–F3 fibrosis [109]. After multivariate analysis, the model included age, BMI, AST/ALT ratio, platelet count, hyperglycaemia and albumin as significant factors. The proposed formula is shown [110] in the equation: NFS = −1.675 + 0.037 × age (years) + 0.094 × BMI (kg/m^2^) + 1.13 × impaired fasting glycemia (IFG) or diabetes (yes = 1, no = 0) + 0.99 × AST/ALT ratio − 0.013 × platelet (×109/L) − 0.66 × albumin (g/dL). A score below −1.455 would be inconsistent with significant fibrosis, whilst a score above 0.676 can diagnose advanced fibrosis. The model was found to have a PPV and an NPV of 90% and 93%, respectively. NFS provides 82% sensitivity, 98% specificity and an AUROC of 0.88 (95% CI, 0.85–0.92). The authors validated their results in a separate group and reported 75% accuracy in excluding or confirming severe fibrosis. Therefore, with this model, only the patients with indeterminate results, i.e., 25% of patients, would require a liver biopsy. In a meta-analysis of 37 studies, Mózes et al. reported the AUROC of NFS to be 0.73 [110]. One of the main limitations of the test is how BMI might be interpreted differently in different ethnic groups. NFS may overestimate the level of fibrosis in morbidly obese patients with NAFLD. Moreover, the test appears to have reduced specificity with advancing age, therefore, different cut-offs may have to be used for patients aged ≥ 65 years and with BMI > 40 kg/m^2^ [111]. Both the NFS and the Fib-4 test cannot always yield binary outcomes and will therefore have an “indeterminate” range. Patients in this category will require second-line testing. Another limitation is the reduced accuracy for detecting earlier fibrosis stages. The use of NFS as a non-invasive way of estimating the presence of advanced fibrosis has been recognised by both the American and European Liver societies [3,4]. The NFS can therefore be used to risk stratify patients, dictate the need for second-line testing and help clinicians and commissioners decide whether further investigations on referral to hepatology services are indicated [33].

The Fibrosis 4 score (Fib-4) was initially developed to assess fibrosis in patients co-infected with HCV/HIV in a multicentre study in 95 centres from 19 countries [112]. The formula for the score is: Fib-4 = Age (years) × AST (U/L)/[PLT(109/L) × ALT1/2 (U/L)]. The AUROC of the model was 0.765. The suggested lower cut-off of the test of <1.45 provided an NPV for detecting advanced fibrosis (Ishak 4–6) of 90%. A cut-off of >3.25 conferred a PPV of 65%. The sensitivity and specificity of the test were found to be 70% and 97%, respectively. Further studies confirmed good predictive accuracy for HCV [112,113] and NAFLD [114]. It is highly sensitive for excluding advanced fibrosis (F ≥ 3). Angulo et al. were able to demonstrate that Fib-4 can be used as a marker to predict the risk of liver-related complications, risk of death and need for liver transplantation [115]. Raised Fib-4 has also been associated with the risk of developing hepatocellular carcinoma in patients with alcohol-related liver disease [116]. One of the potential limitations of the test is the possible reduced specificity with advancing age, therefore, different cut-offs may have to be used for patients aged ≥ 65 years [111]. Similarly, NFS and Fib-4 could be used as a screening tool in clinical practice, as it is relatively affordable and easy to obtain and can accurately exclude patients with advanced fibrosis. Likewise, the use of NFS and Fib-4 as a non-invasive way of estimating the presence of advanced fibrosis has been recognised by both the American and European liver societies, and it can inform the way patients with NAFLD will be managed in the healthcare system [3,4]. The use of simple non-invasive tests, such as NFS, Fib-4, APRI and BARD, could also be considered for wider population screening to predict long-term outcomes [117].

The AST/ALT ratio was initially developed in a cohort of 139 patients with chronic hepatitis C infection [118]. With fibrosis, the AST increases or remains stable, whilst the ALT falls. In normal subjects, the ratio is ~0.8. The ratio of AST/ALT was found to correlate positively to the degree of fibrosis but not the disease activity grade. The studied endpoint was the ability of the test to predict cirrhosis. The test was found to have a PPV and NPV of 100% and 80.7%, respectively, when a cut-off ratio of >1 was used. The sensitivity and specificity were reported as 53.2% and 100%, respectively [118]. Many subsequent studies also focused on patients with HCV and autoimmune diseases such as PBC, and the results have been inconclusive as to the ability of AST/ALT to predict the presence of cirrhosis [119,120,121]. The AST/ALT is simple to calculate but has a low diagnostic accuracy, with an AUROC of 0.66–0.74. In patients with NAFLD and NASH, the AST/ALT ratio is often <1 [122]. However, it should be noted that with progressive fibrosis, AST levels rise and ALT revels drop, which can result in an increasing ratio [122,123]. Patients with ALD disease will often have an AST/ALT ratio of >1.5.

Just like the AST/ALT ratio, the AST/PLT ratio index (APRI) was developed from a study of patients with HCV to predict the presence of advanced fibrosis (Ishak score > 3)/cirrhosis [124]. APRI can be calculated as: APRI = (AST elevation/PLT count) × 100. Wai et al. examined the histological samples of 192 treatment-naïve HCV patients and validated their results in an additional cohort [124]. The AUROCs of the test for predicting significant fibrosis and cirrhosis were 0.87 (95% CI, 0.79–0.95) and 0.93 (95% CI, 0.85–1.0), respectively. In their meta-analysis, Lin et al. looked at data from 40 studies and 8739 patients with HCV [125]. The AUROCs for the diagnosis of significant fibrosis, severe fibrosis and cirrhosis were 0.77, 0.80 and 0.83, respectively. With an APRI score of >1.0, the test was 61% sensitive and 64% specific for severe fibrosis. With the same score threshold, the model was 76% sensitive and 72% specific for patients with cirrhosis [125]. Even though the test was initially developed for patients with viral hepatitis, its usefulness has been extrapolated for a patient with NAFLD as well. In patients with NAFLD, the APRI score could be useful in the diagnosis of fibrosis, as it appears to increase with a higher METAVIR score [126]. High APRI scores have also been associated with higher cardiovascular risk in patients with metabolic syndrome [127]. In their recent systematic review, Lee et al. identified and pooled the data of 10 studies looking at APRI in patients with NAFLD [128]. Whilst APRI was shown to have a good ability to prognosticate the occurrence of liver-related adverse outcomes and stage of fibrosis, it appears to underperform when it comes to predicting liver-related death.

Following multivariable analysis, Ratziu et al. proposed the BAAT score, which includes four independent variables (BMI, age, ALT and TGs) and has been shown to predict the presence of septal fibrosis in overweight patients [129]. Patients are scored based on BMI (≥28 = 1, <28 = 0), age at liver biopsy (≥50 years = 1; <50 = 0), ALT (≥2N = 1, <2N = 0) and serum triglycerides (≥1.7 mmol/L = 1, <1.7 = 0). The score ranges from 0–4 and was found to demonstrate an AUROC of 0.84 [129]. In patients whose score was 0 and 1, the sensitivity of the BAAT score was 100%, and the specificity was 47%. The PPV and NPV were 45% and 100%, respectively [129].

Harrison et al. collected retrospective data from more than 800 patients from two large American tertiary centres in the US [130]. The study identified three variables that were associated with a higher risk of hepatic fibrosis. The three parameters were developed into a composite score, i.e., the BARD score with a maximum score of 4 (BMI ≥ 28 = 1 point, AST/ALT ratio ≥0.8 = 2 points and presence of diabetes = 2 points). The authors identified patients with a BARD score of 2–4 and used logistic regression analysis to predict the risk of advanced hepatic fibrosis, i.e., Metavir F3–4. The AUROC was 0.81, and the PPV and NPV were 43% and 96%, respectively. BARD score’s main limitation is the high false positivity, which is based primarily on the overestimation of BMI and, secondarily, the presence of diabetes [130].

The ADPAT (age, diabetes, PRO-C3 and PLT) score was developed as a biomarker for advanced fibrosis in patients with NAFLD. Daniels et al. studied outcomes in 431 patients with biopsy-confirmed NAFLD and showed a good diagnostic accuracy of ADAPT with an AUROC of 0.86 (95% CI, 0.79–0.91) [131]. With a score of >6.3287, the test had a PPV and an NPV for advanced fibrosis of 48% and 97%, respectively. The sensitivity and specificity of ADAPT were 91% and 73%, respectively.

The Nippon score was developed following an evaluation of the outcomes of 182 Japanese patients with biopsy-proven NAFLD [130]. Gender, age ≥ 60 years old, presence of T2DM and hypertension were identified as independent factors for advanced liver fibrosis. Nippon score has demonstrated 84% sensitivity, 82% specificity and 0.78 AUROC [130].

A summary of the biochemical diagnostic tools is presented in Table 1.

## 3. Imaging Biomarkers

The use of non-invasive imaging modalities plays an important part in the diagnosis of patients with NAFLD [132]. Over the last two decades, there has been significant development in the evaluation of NAFLD by non-invasive imaging, and novel techniques with high accuracy are now widely available [132]. According to the recently developed clinical care pathway by the American Gastroenterological Association (AGA), imaging modalities in combination with biochemical indices for NAFLD offer significant improvements in the diagnosis of NAFLD and screening for advanced fibrosis [29]. In this section, we discuss the various non-invasive modalities being available for the diagnosis of NAFLD, as well as their advantages and limitations.

### 3.1. Ultrasonography (USS)

The use of conventional B-mode ultrasound technology is well established in liver imaging, largely because of its simplicity, wide availability and cost effectiveness compared to other imaging techniques [133]. Furthermore, ultrasonography is considered a safe, non-invasive and well-tolerated procedure, as it avoids exposure to ionising radiation and is often performed without the need for intravenous access or use of intravenous contrast [133,134]. Ultrasound-based imaging is the preferred first-line modality when screening patients with incidental derangement of liver enzymes [3,135]. The European Association for the Study of the Liver (EASL), the European Association for the Study of Diabetes (EASD) and the European Association for the Study of Obesity (EASO) released a joint statement strongly recommending ultrasound as first-line imaging for NAFLD, based on high-quality Level 1 evidence [3]. Ultrasound is also the most frequently used technique when evaluating patients for hepatic steatosis, which is characterised by a hyperechoic appearance, often referred to as a bright liver [136]. It is reliable when >33% of hepatocytes are steatotic and produces an appearance of diffuse, smooth, tightly packed echo pattern that is quite distinctive of a steatotic liver and is reproducible on ultrasound [136,137,138].

High diagnostic accuracy of the USS for the diagnosis of NAFLD has been demonstrated by several studies [135,137,139]. A meta-analysis by Hernaez et al., including 4720 patients, compared ultrasound findings to those of liver biopsy and found that ultrasound assessment for moderate to severe hepatic steatosis had a sensitivity of 85%, a specificity of 94%, a positive likelihood ratio of 13.3, a negative likelihood ratio of 0.16 and an AUROC of 0.93 [135]. This was similar to Palmentieri et al., who compared bright liver echo pattern to histology in detecting moderate and severe steatosis (>30% fatty infiltration) and reported the sensitivity, specificity, positive predictive and negative predictive values to be of 91%, 93%, 89% and 94%, respectively [139].

The extent of hepatic steatosis can be qualitatively graded using a 4-point scale ranging from Grade 0 (normal appearance) through to Grade 3 (severe steatosis) [137]. However, the quantification of steatosis using ultrasound is subjective, as it is based on the qualitative visual impression of the operator and is therefore less accurate at detecting steatosis at the milder spectrum of the disease [28,137,140]. This was evident when ultrasound findings were compared to the gold-standard liver biopsy in a double-blinded prospective study by Dasarathy et al. [28]. The authors demonstrated high specificity (90%) and sensitivity (100%) when there was at least 20% fatty infiltration, but the sensitivity was significantly reduced with a high false-negative rate when there was less than 20% fat [28]. Diagnostic accuracy of the ultrasound is also limited in patients with fibrosis, chronic liver dysfunction, high body mass index (BMI > 40 kg/m^2^) or in the presence of ascites [141,142,143]. These limitations can be partially overcome with the use of semi-quantitative measures, such as the ultrasonographic fatty liver indicator (US-FLI score) [144]. The US-FLI uses sonographic metrics (hepato-renal contrast, posterior attenuation, blurring of portal veins and hepatic veins, limited visualisation of the gallbladder wall, limited visualisation of the diaphragm and areas of focal fatty sparing) to generate a score of mild, moderate and severe steatosis. Studies demonstrate excellent inter-observer reproducibility and good differentiation of severity, with high specificity (90%) and sensitivity (90%) even in mild steatosis (>10% fatty infiltration) [145,146]. The US-FLI score has also been found to differentiate steatosis from NASH in the obese population, especially when the score is ≤4 with a negative predictive value of 88% and sensitivity of 91% for absence of NASH [144].

### 3.2. Computed Tomography (CT)

CT scans are widely available, allow rapid acquisition of data and are less costly than magnetic resonance imaging (MRI) scans [147]. The assessment of hepatic steatosis on CT is most commonly performed using the attenuation difference between the liver and spleen on unenhanced CT scans [147]. Using the spleen as an internal control mitigates for the variability in attenuation measurement of liver parenchyma between different CT scanners and different reconstruction algorithms [141,142,148]. Moreover, the use of unenhanced scans is preferable, as contrast injection protocols and scan delays can affect the attenuation of the liver parenchyma [142]. This technique has high diagnostic accuracy, with a reported sensitivity and specificity of 64–90% and 95–100%, respectively, in detecting moderate to severe steatosis [148,149]. However, CT scanning is less reliable in the detection of mild steatosis [150,151,152]. The area under the ROC curve has been reported as 0.74 and 0.93 at detecting mild (>5%) and moderate (>33%) steatosis, respectively [152]. Additionally, iron deposition and the presence of iodine-containing material, including contrast, increase hepatic attenuation and confound the assessment of hepatic steatosis on standard CT scanners [153]. These factors, in combination with the use of radiation, make CT scanning an unsuitable imaging modality for the primary assessment and follow-up of patients with NAFLD [150]. Newer techniques, such as dual-energy CT, have the potential to provide improved diagnostic accuracy for CT assessment of steatosis, although thus far studies show comparable results to ultrasound and single-energy CT scans [154,155]. However, CT scans can be useful in the assessment of steatosis in living donor candidates, as it also allows accurate assessment of hepatic vasculature [156].

### 3.3. Conventional Magnetic Resonance Imaging (MRI)

Various MRI techniques are available with the conventional MRI, also known as in-phase (IP) and opposed-phase (OP) imaging, which can detect hepatic steatosis by exploiting the difference in resonance frequencies between water and fat proton signals [157]. By utilising fat suppression methods (T1 and T2-weighted echo sequences) or acquiring images at echo times when water and fat signals are in phase or opposed phase, hepatic steatosis can be qualitatively visualised [158]. A meta-analysis by Wang et al. reported that the use of MRI had a sensitivity of 82%, specificity of 87% and AUROC of 0.95 in the diagnosis of NAFLD and the stage of the disease [159]. However, some factors can confound the signal, making MRI not suitable for accurate qualitative assessment of hepatic steatosis [158]. These confounders include T1-related bias (when water and fat have different T1 values in a T1 acquisition), T2 decay (images are acquired at different echo times), the spectral complexity of fat, noise-related bias and even temperature [157,158]. These confounders can be mitigated with chemical-shift-encoded MRI, which exploits the chemical shift between water and fat resonance frequencies, such as in MR proton density fat fraction (MR-PDFF) images and spectroscopy (MRS) [158].

### 3.4. MR-Proton Density Fat Fraction (MRI-PDFF)

The MRI-PDFF is a novel image-based biomarker that maps the entire liver within seconds as a ratio of proton density from TGs to the proton density of TGs and water combined. It has been shown to demonstrate high accuracy at detecting all grades of steatosis, with an AUROC of 0.99, and has been found to be superior to the controlled attenuation parameter (CAP), which is discussed later [160,161]. A longitudinal study involving NAFLD by Noureddin et al. found that MRI-PDFF was more sensitive than the histology-determined steatosis grade in quantifying changes in liver fat content, suggesting that MRI-PDFF could be used for quantitative assessment of steatosis [162]. A recent meta-analysis by Gu et al., including six studies and 635 patients in total, showed high sensitivity and specificity of MRI-PDFF for classifying steatosis grades, indicating its important diagnostic value (Table 2) [163]. Although among the non-invasive imaging techniques MRI-PDFF has the highest accuracy in the diagnosis of steatosis in NAFLD patients, its use is still limited to research due to its high cost and the special equipment and training required for its use [164].

### 3.5. Liver MultiScan

Recently, an MRI-PDFF technique known as iterative decomposition of water and fat with Echo Asymmetry and Least-squares estimation (IDEAL) has been further developed with regard to data processing [165]. These refinements have led to a software modality called Liver MultiScan (LMS-IDEAL), which is used to quantitatively characterise the hepatic parenchyma in terms of fat composition, iron, inflammation and fibrosis [165]. LMS (Perspectum Diagnostics, Oxford, UK) is compatible with various MRI machines, with the advantage of no need for contrast [166]. Similarly to MRI, Liver MultiScan should not be used in patients with metal implantable devices. The diagnostic potential of LMS has been assessed by a few prospective studies, which reported that the Liver Inflammation and Fibrosis (LIF) score demonstrated a significant positive correlation with the histological findings from liver biopsies in NAFLD patients [167,168,169,170]. Additionally, the LIF score is proposed to be used to differentiate between different stages of liver disease with a cut-off of 3.0 to show 91% sensitivity and 73% specificity for the diagnosis of liver cirrhosis [170]. Although evidence is still limited, LMS is considered a promising non-invasive tool for the diagnosis of NAFLD, and it is expected to reduce the number of liver biopsies needed by 16% if used alone and by 66% when used in combination with liver elastography [171]. LMS is increasingly used in research studies, and ongoing clinical trials are anticipated to assess its use as a non-invasive diagnostic modality in NAFLD [172].

### 3.6. H-Magnetic Resonance Spectroscopy (1H-MRS)

1H-MRS imaging is another and earliest reported MRI technique for quantifying hepatic steatosis [158]. 1H-MRS measures proton signals within liver tissue and generates multiple peaks from chemicals or metabolites, which are expressed as a shift in frequency relative to water as standard [173]. 1H-MRS has been found to be a safe and reproducible method for the quantification of fat in both liver lobes [174]. However, it is limited largely by motion from respiration and cardiac pulsation, causing a low signal to noise ratio [173]. To overcome this limitation, manual data processing is required, which is a complex and time-consuming process, making this technique not widely available [173]. 1H-MRS has repeatedly been shown to have excellent diagnostic performance for the diagnosis and quantification of hepatic steatosis [175,176]. When compared to other imaging modalities and using liver biopsies as a reference standard, a large meta-analysis by Bohte et al. demonstrated superior overall performance for MRS with an overall sensitivity of 73–91% and a specificity of 92–96% [151]. This advantage is especially prominent when evaluating patients with mild steatosis, which is a limitation for both ultrasound and CT. MRS was found to have a sensitivity of 83–89% and a specificity of 94–96% in patients with a mild steatotic disease [151]. Moreover, in contrast to the inter-observer variability seen with ultrasound scans, MRS has better reproducibility with less than 1% standard deviation from repeat measurement of PDFF [177].

Although histopathological evaluation remains the gold standard for the evaluation of hepatic steatosis, several studies have found that MRI-PDFF was better correlated with actual fat content in comparison to MRS [162,178,179]. Furthermore, MRS assuages the limitations of liver biopsies, including the need for an invasive procedure with the associated risk of complications and the inter- and intra-observer variability of histopathological assessment [162,180]. Consequently, MRS is often considered an alternate reference standard for detecting and quantifying hepatic steatosis [142]. However, similar to liver biopsies, MRS uses a small sample size, and this can be misleading in patients with uneven fatty changes. MRS scans are also expensive and not currently universally accessible. As a result, its use is often limited to research [150].

### 3.7. Liver Elastography Modalities

Liver elastography consists of the basic principle of passing an acoustic impulse through the tissue that causes minimal displacement of tissue and the formation of shear waves in liver tissue, which move faster in the presence of stiffness (i.e., cirrhosis) [181]. Elastography can be MRI based or ultrasound based [181].

#### 3.7.1. Magnetic Resonance Elastography (MRE)

MRE had been approved by the Food and Drug Administration in 2009 and is currently one of the most accurate non-invasive methods for quantitative assessment of liver stiffness and therefore for the diagnosis of liver fibrosis [182]. MRE is highly reproducible and it is not affected by other factors, such as obesity, sex and aetiology of chronic liver disease [181,183]. Electrogram protocols are included in conventional MRI scanners, making MRE a widely available imaging modality [184]. However, similarly to other MRI-based techniques, MRE is hindered by respiratory or cardiac motion artefacts [184]. Several systematic reviews and meta-analyses have concluded that MRE may have the highest diagnostic accuracy for NAFLD fibrosis, independent of BMI and inflammation [185,186]. Two recent meta-analyses demonstrated that MRE has high diagnostic accuracy for the detection of any fibrosis and shows excellent ability to identify significant fibrosis and cirrhosis (Table 2) [186,187]. From a recent multicentre study, a proposed cut-off of 4.39 kPa was found to distinguish liver cirrhosis with an AUROC of 0.92 [188]. The AASLD guidelines highlight the role of MRE to differentiate between various degrees of fibrosis and propose this imaging modality as a useful tool for the diagnosis of NAFLD patients with advanced liver fibrosis [2].

#### 3.7.2. Vibration-Controlled Transient Elastography (VCTE)

VCTE is a USS-based method of assessing liver stiffness by measuring shear wave velocity when a sound wave passes through the liver. A common device used is the Fibroscan^®^ and, according to NICE, EASL and AASLD guidelines, it is recommended as the first test for assessment of advanced liver fibrosis in primary care [2,189]. VCTE is widely available, cost effective and well tolerated by patients [190]. However, its accuracy is limited by factors such as obesity, ascites, hepatitis, T2DM and cholestasis, which may lead to unreliable and non-reproducible results [190,191]. The role of VCTE in the diagnosis of advanced fibrosis and cirrhosis is more widely established in viral hepatitis patients, with up to 96% diagnostic accuracy [192]. Two recent meta-analyses have evaluated the diagnostic performance of VCTE in NAFLD in comparison with other non-invasive tests [191,193]. Hsu et al. reported that VCTE had good diagnostic accuracy for detection of fibrosis, with an AUROC ranging between 0.82 to 0.84; however, MRE was found to demonstrate significantly better diagnostic accuracy in detecting fibrosis at all stages in NAFLD patients [193]. Mozes et al. conducted the largest meta-analysis to date regarding the diagnostic accuracy of VCTE in comparison with other non-invasive scores (FIB-4, NFS, APRI, AST/ALT) for identifying advanced fibrosis and cirrhosis. VCTE, with 77% sensitivity, 78% specificity and an AUROC of 0.85, was found to perform better than other scores with proposed cut-offs <7.1 kPa and ≥14.1 kPa (*p* < 0.0001). Interestingly, Mozes et al. also reported that the sensitivity and specificity for advanced fibrosis were increased when sequential combinations were performed between VCTE and the rest of the non-invasive scores, suggesting that liver biopsies could be decreased from 33% to 19% [191]. EASL guidelines recommend that VCTE should not be used alone for detection of advanced fibrosis and cirrhosis [194].

#### 3.7.3. Controlled Attenuation Parameter (CAP)

CAP is another non-invasive method that is based on VCTE to detect liver steatosis and implemented on Fibroscan [190]. CAP/TE enables the quantification of fat accumulation in the liver by measuring the level of ultrasound attenuation caused by liver fat [195]. CAP offers the advantage of being widely available and can simultaneously evaluate liver stiffness [196]. Additionally, CAP has been shown to be able to differentiate between different steatosis grades without being influenced by the presence of fibrosis or cirrhosis [190,196]. A recent meta-analysis by Cai et al., which included 1936 ALD/NAFLD patients, reported high diagnostic accuracy of CAP for detecting steatosis grade ≥S1 at an average threshold of 272 dB/m with an AUROC of 0.90 [197]. However, the accuracy was noted to be reduced with the progression to steatosis grades with an AUROC of 0.83 and 0.79 for steatosis grade ≥S2 and ≥S3, respectively. Sub-group analyses showed that the diagnostic accuracy of CAP was less in patients with high BMI (≥28) [197]. To overcome this limitation, the XL probe has recently become available for the CAP/TE modality, allowing it to be used in the obese NAFLD patient group [198]. A recent meta-analysis of XL probe use in 1050 patients, 89% of whom had NAFLD, gave AUROC of 0.82 for S0 vs. S1 to S3, and 0.754 for S0/S1 vs. S2/3 [198]. The authors suggest that the use of CAP should be limited to the screening of NAFLD, rather than grading [198]. When applied to fibrosis, the XL probe used in patients with a BMI >30 showed increased diagnostic utility and decreased failure rate when compared to the medium probe [199].

#### 3.7.4. Point Shear Wave Elastography (pSWE)

Acoustic radiation force impulse (ARFI), known as point shear wave elastography (pSWE), is a non-invasive ultrasound-based method that uses acoustic radiation impulse to induce shear waves at a single point in the liver, therefore assessing liver stiffness [181]. It has been shown to demonstrate high reproducibility and repeatability without being affected by the presence of inflammation or ascites [181]. Studies have shown that pSWE demonstrates moderate diagnostic accuracy in detecting liver fibrosis in NAFLD patients with an AUROC of 0.89 [200]. A meta-analysis by Jang et al. reported high diagnostic accuracy of pSWE for the detection of advanced fibrosis and cirrhosis in NAFLD, with an AUROC of 0.94 and 0.95, respectively [201]. Similar findings were reported by another meta-analysis by Xiao et al., which showed that among various non-invasive tests (FIB-4, BARD score, NFS, Fibroscan), pSWE and MRE demonstrated the highest diagnostic accuracy for advanced fibrosis with an AUROC of 0.95 and 0.96, respectively [185]. Similarly, with pSWE, 2D SWE uses acoustic radiation force to displace the liver tissue at multiple sites, measuring liver stiffness [202]. It offers similar advantages to pSWE in terms of reproducibility, patient acceptance and repeatability [181]. Two-dimensional SWE has been reported to demonstrate higher sensitivity than pSWE in detecting each stage of liver fibrosis in patients with chronic liver disease. However, evidence on the role of pSWE as a diagnostic modality in NAFLD is still limited, as no meta-analysis has been available to date [203]. A recent prospective study involving 114 NAFLD patients showed a moderate to high diagnostic accuracy of 2D SWE with AUROCs of 0.84, 0.88 and 0.93 for detection of fibrosis stages 2, 3 and 4, respectively [204]. In this study, VCTE demonstrated better accuracy than 2D SWE (*p* = 0.03). Future studies are needed to evaluate the role of 2D SWE as a diagnostic modality in NAFLD [204].

A summary of the imaging modalities discussed above is presented in Table 2.

**Table 2:** Non-invasive imaging diagnostic modalities in NAFLD.

Method	Description	NAFLD Stages	Accuracy (SS/SP/AUROC)	Advantages	Disadvantages	Refs
Ultrasound	Fat deposition increases the amount of beam scattering, leading to increased echogenicity (bright liver)	Steatosis	High (85%/94%/0.93)	No radiation, Low cost Widely available	Operator dependent ↓ accuracy in obesity, advanced fibrosis, cirrhosis	[28,135,143]
CT	Assessment is performed using the attenuation difference between the liver and spleen on an unenhanced CT scan	Severe steatosis Cirrhosis	High (90%/100%/0.93)	Widely available Low cost Useful in assessing liver vasculature	High cost ↓ accuracy in mild steatosis Radiation	[150,151,152,153,154,155]
Conventional MRI	Difference in resonance frequencies between water and fat proton signals	Steatosis Fibrosis	High (87%/82%/0.95)	No radiation	High cost Not widely available Accurate only in cirrhosis	[157,158,159,205]
MRI-PDFF	Ratio of proton density from TGs to the total proton density of TGs and water	All grades of steatosis	Very high (96%/100%/0.99)	Not affected by confounding factors (e.g., obesity) Simultaneous assessment for carcinoma and steatosis	High cost Not widely available ↓ accuracy in inflammation, iron overload Not suitable for patients with implantable devices	[134,162,163]
LMS	Iterative Decomposition of water and fat with Echo Asymmetry and Least-squares estimation (IDEAL) plus MRI data processing software	All grades of steatosis	High (91%/73%)	Similar as MRI-PDFF Can distinguish iron composition	High cost Not widely available	[168,169,170,172]
1H-MRS	Generates peaks from proton signals from chemicals or metabolites within liver tissue	Mild steatosis	High (71–93%/92–96%)	Very useful in mild steatosis, amounts of fat as low as 0.5% can be detected High reproducibility	Complex and laborious data analysis Uses small liver sample ↓ accuracy in cirrhosis Limited by respiration and pulsating movements Not widely available	[134,151,173,174,177]
MRE	Characterises the biomechanical properties of tissues, such as stiffness, through the application of mechanical shear waves to the tissues	Fibrosis Stage 1 Stage 2 Stage 3 Stage 4	High (75%/77%/0.86) (79%/81%/0.87) (83%/86%/0.90) (88%/87%/0.91)	High accuracy High reproducibility Not affected by obesity/ascites	Limited by respiration and pulsating movements Similar to MRI limitations Not widely available	[182,185,186,187,206]
VCTE	Measures shear wave velocity of liver tissue when a sound wave passes through the tissue and assesses liver stiffness	Advanced fibrosis	Moderate (77%/78%/0.85)	Widely available in primary care Cost effective Well tolerated	Limited by obesity, T2DM, ascites ↓ reliability in steatosis ↓ accuracy in early fibrosis	[191,193,207]
CAP/TE	Measures the extent of ultrasound attenuation by hepatic adipose tissue based on TE performed alongside	Steatosis ≥S1 ≥S2 ≥S3	Moderate (84%/83%/0.9) (83%/71%/0.83) (78%/62%/0.78)	Widely available in primary care Cost effective Provides immediate assessment of steatosis, as well as liver stiffness	Limited by obesity, T2DM, ascites ↓ accuracy in early fibrosis ↓ reliability in distinguishing between steatosis grades	[181,195,196,197,198,199]
ARFI-pSWE	Induces shear waves in the liver at a single site using acoustic radiation impulse and assesses liver stiffness	Advanced fibrosis	Moderate/High (50%/84%/0.95)	High repeatability and reproducibility	Limited by obesity ↓ accuracy in steatosis and cirrhosis	[181,185,200,201]
2D SWE	Induces shear waves in the liver at multiple sites using acoustic radiation impulse and assesses liver stiffness	Fibrosis	Moderate (53%/90%/0.72)	High repeatability and reproducibility	Limited by obesity ↓ accuracy in steatosis and cirrhosis	[203,204]

AUROC: Area Under the Receiver Operator Curve, ARFI-pSWE: Acoustic Radiation Force Impulse—Point Shear Wave Elastography, CAP: Controlled Attenuation Parameter, CT: Computed-Tomography, MRI: Magnetic Resonance Imaging, MRE: Magnetic Resonance Elastography, PDFF: Proton Density Fat Fraction, LMS: LiverMultiScan, T2DM: Type 2 Diabetes Mellitus, TE: Transient Elastography, TGs: Triglycerides, SS: Sensitivity, SP: Specificity, VCTE: Vibration-Controlled Transient Elastography, 1H-MRS: Proton Magnetic Resonance Spectroscopy, 2D SWE: 2D Shear Wave Elastography.

## 4. Omics-Related Diagnostic Research Technologies

**Figure 2:**
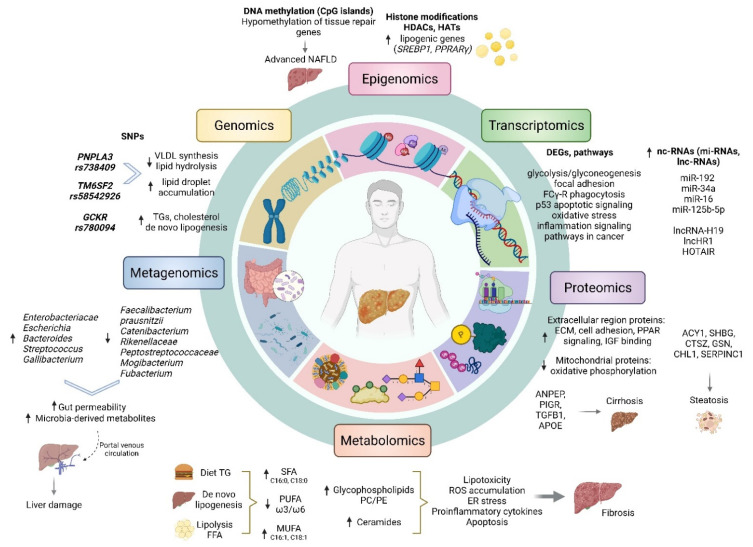
An overview of the omics modifications in NAFLD pathogenesis and their potential role as diagnostic biomarkers (created with BioRender.com, accessed on 21 December 2021). ACY1: Aminoacylase 1, ANPEP: Alanyl Aminopeptidase, APOE: Apolipoprotein E, CHL1: Cell Adhesion Molecule L1 like, CTSZ: Cathepsin Z, DEGs: Differentially Expressed Genes, ECM: Extracellular Matrix, ER: Endoplasmic Reticulum, FCγ-R: Fcγ Receptors, FFA: Free Fatty Acid, GSN: Gelsolin, GCKR: Glucokinase Regulatory Gene, HDACs: Histone Deacetylases, HATs: Histone Acetyltransferases, HOTAIR: HOX Transcript Antisense RNA, IGF: Insulin-like Growth Factor, lncRNAs: long non-coding RNAs, mi-RNAs: microRNAs, MUFA: Monounsaturated Fatty Acid, nc-RNAs: non-coding ribodeoxynucleic acids, PPRARγ: Peroxisome proliferator-activated receptor gamma, PIGR: Polymeric Immunoglobulin Receptor, PC: Phosphatidylcholine, PE: Phosphatidylethanolamine, PUFA: Polysaturated Fatty Acid, PNPLA3: Patatin-like phospholipase domain-containing 3, ROS: Reactive Oxygen Species, SREBP1: Sterol Regulatory Element-Binding Protein 1, SERPINC1: Serpin family C member 1, SFA: Saturated Free Acid, SHBG: Sex Hormone Binding Globulin, SNP: Single-Nucleotide Polymorphism, TGFB1: Transforming Growth Factor Beta 1, TG: Triglycerides, TM6SF2: Transmembrane 6 Superfamily Member 2, VLDL: Very-Low Density Lipoprotein.

Since the sequencing and mapping of the human genome, new technologies have enabled the acquisition of molecular measurements within tissues. Such technologies, which are associated with measuring molecules in a high-throughput method, are called “omics” and include, genomics, epigenomics, transcriptomics, metabolomics, lipidomics and metagenomics [208]. This section gives an overview of the diagnostic role of the omics approaches in NAFLD (Figure 2).

### 4.1. Genomics

The development of high-throughput sequencing technologies, oligonucleotide arrays, as well as advancements in computational biology and bioinformatics, has led to a rapid increase in multiple genome-wide association studies (GWAS), which have improved our understanding of genetic biomarkers for the diagnosis and pathogenesis of NAFLD [207]. To date, the National Human Genome Research Institute (NHGRI)-GWAS catalogue contains 24 GWAS studies reporting more than 100 gene variants related to the NAFLD trait [209]. For this review, the most common risk variants in NAFLD pathogenesis will be discussed.

The Patatin-like phospholipase domain-containing 3 gene (PNPLA3) located in 22q13 is expressed in the liver and adipose tissue and encodes for the adiponutrin protein, which exhibits lipase activity towards triglycerides and retinyl esters in hepatocytes and hepatic stellate cells, respectively [210]. The PNPLA3 variant rs738409 C>G is a non-synonymous Single-Nucleotide Polymorphism (SNP), which results in isoleucine (I) to methionine (M) substitution at position 148 (I148M) [210,211]. The PNPLA3 I148M substitution causes loss of function in the enzymatic activity of the adiponutrin protein, leading to the accumulation of lipid droplets in hepatocytes [210]. A GWAS in a multi-ethnic population-based sample study by Romeo et al. first reported a robust association between rs738409 C>G p.I148M and increased hepatic fat levels and liver inflammation, thus contributing to susceptibility to NAFLD [211]. Further GWAS studies have replicated the correlation of rs738409 C>G p.I148M with steatosis, fibrosis and NAFLD disease severity [212,213,214]. A recent meta-analysis suggested that patients with GG genotype are 105% more likely to develop NAFLD than patients who had other genotypes (OR 2.5, 95% CI, 1.64–2.56) [215]. Rs738409 is now considered an established genetic biomarker for NAFLD, with G-allele carriers also being at risk of liver failure and liver-related fibrosis mortality, as well as developing hepatocellular carcinoma [216].

Glucokinase regulatory gene (GCKR) is located in chromosome 2p23, expressed in liver tissue, and its protein inhibits glucokinase in hepatocytes [217]. Rs780094 (T>C) and rs1260326 (T>C/T>G) are two variants that have been identified to be associated with increased risk of NAFLD in various genetic models, with the former variant being more common [218,219]. A proposed mechanism by which rs780094 may predispose to NAFLD is that the variant protein does not exert inhibitory effect on glucokinase, therefore, there is an activation of de novo lipogenesis, TG and cholesterol synthesis [220]. On the contrary, some studies report no association between rs780094 and NAFLD risk, indicating that there may be population differences, highlighting the need for further research evidence to be obtained with regard to GCKR and NAFLD risk [217].

Transmembrane 6 superfamily member 2 (TM6SF2) is located in 19p13 and is expressed mainly in the liver, small intestine and renal tissues [221]. TM6SF2 encodes a protein involved in the regulation of very-low-density lipoprotein (VLDL) secretion and synthesis from hepatocytes [221]. The rs58542926 C>T non-synonymous polymorphism leads to a Glutamic acid (E) to Lysine (K) substitution at position 167, resulting in a loss of function and dysregulation of the VLDL synthesis [222]. In vitro models have associated TM6SF2 E167K variant with decreased VLDL secretion, and human studies have observed the presence of phosphatidylcholine (PC) deficiency, thus impeding VLDL synthesis in rs58542926 carriers [221]. The first exome-wide association study (EWAS) showed that the TM6SF2 E167K variant was associated with higher ALT and lower LDL-cholesterol, TGs and ALP plasma levels in different populations [223]. Additional, mechanistic in vivo experiments showed that a knockdown of the TM6SF2 gene resulted in a two-fold increased liver TG content and 50% decreased VLDL secretion in mice [223]. A meta-analysis by Pirola et al. illustrated a positive association between rs58542926 (EK and KK genotype) and NAFLD risk (OR 2.13, 95% CI, 1.36–3.30), as well as a significant increase in TGs and liver fat content [224].

Membrane-bound O-acetyltransferase domain-containing 7 (MBOAT7) is a gene encoding for an enzyme involved in the Lands cycle of remodelling the membranes through deacylation and reacylation, thus regulating the production of free arachidonic acid, which triggers liver inflammation and fibrosis [225]. MBOAT7 upregulation has been shown to be associated with anti-inflammatory processes, and the rs641738 C>T SNP has been found to lead to downregulation of the MBOAT7 mRNA and protein in hepatocytes [222,225]. By genotyping a large multi-ethnic population, Mancina et al. found that the rs641738 variant was associated with increased risk of hepatic TG content (OR 1.2, 95% CI, 1.05–1.37, *p* = 0.006), a higher degree of liver steatosis (OR 1.18, 95% CI, 1.07–1.91, *p* = 0.050) and advanced fibrosis (OR 1.30, 95% CI, 1.06–1.70, *p* = 0.012) [226]. The causative relationship of MBOAT7 with fatty liver has been experimentally confirmed by several in vitro and in vivo studies, showing that MBOAT7 loss leads to steatosis development, hepatic fibrosis and the formation of lipid droplets in hepatic cells [225].

Several other genes, such as APOB, SERPINA1 and HSD17B13, have been found to be associated with NAFLD [227,228,229,230]. In a large prospective study involving 1139 participants, Wang et al. found that increased APOB levels (>1.17 g/dL) were independently associated with NAFLD in multivariable models adjusted for patient demographics and clinical and biochemical variables (OR 1.631, 95% CI, 1.17–2.26, *p* = 0.001) [227]. SERPINEA1 rs17580 variant was found to be associated with an increased risk of liver fibrosis and cirrhosis in NAFLD (OR 3.42, *p* = 0.01 and OR 2.59, *p* = 0.02) Basyte-Bacevice1. Additionally, the SERPINA1 rs28929474 variant has been reported to be associated with portal hypertension (OR 2.122, 95% CI, 1.07–4.22, *p* = 0.032) [228]. Conversely, the rs72613567 rs6834314 polymorphisms of HSD17B13 were associated with reduced risk of liver fibrosis (OR 0.37, *p* = 0.03) and NASH in various studies [229,230].

The recent practice of next-generation sequence in patients with NAFLD has linked the mitochondrial DNA (mtDNA) mutation spectrum with disease severity [231]. Sookoian et al. showed that increased mtDNA mutational rate, including mutations in genes of the oxidative phosphorylation (OXPHOS) chain, was observed in the liver of patients with NAFLD in comparison with matched control [232].

Several mtDNA variations have been found to be associated with NAFLD development and severity. In more detail, Mt16249CC was found to be associated with advanced steatosis and hepatic inflammation, Mt16318C with NASH and Mt16129AA with advanced fibrosis [233]. Recently, mutations in the mitochondrially encoded cytochrome B (mtCYB) have been linked with NAFLD by triggering a proinflammatory response and linking oxidative damage to inflammation [234]. Pirola et al., through analysing liver biopsies from NAFLD patients, reported that NASH was associated with significantly higher mtCYB genetic variations compared to simple steatosis [234].

It has become evident that nuclear and mitochondrial gene variability has been linked to NAFLD pathogenesis and progression [233]. GWAS studies have contributed significantly to research towards identifying genetic biomarkers in the diagnosis and prediction of NAFLD. Several studies have attempted to build genetic risk scores as non-invasive genetic biomarkers. Increased polymorphism score of PNPLA3, GCKR, TM6SF2 and MBOAT7 has been found to be predictive of NAFLD after being adjusted for age, BMI, TG levels and Homeostatic Model Assessment-Insulin Resistance (HOMA-IR) [235]. Despite the numerous genetic variations having been observed in NAFLD, only a few have been validated (PNPLA3, TM6SF2) [3]. Although genotyping of PNPLA3 and TM6SF2 variants is not routinely advised for NAFLD diagnosis, the EASL/EASD/EASO guidelines recommend their assessment in a selected group of patients [3].

### 4.2. Epigenomics

Epigenetics is the study of changes in gene expression and phenotypic variation not resulting from alternations in DNA sequence [222]. The most characterised epigenetic modifications include DNA methylation and histone modification [236].

#### 4.2.1. DNA Methylation

DNA methylation is an important epigenetic modification that occurs at the cytosine base within cytosine-phospho-guanine (CpG) dinucleotides, known as CpG islands [237]. CpGs are often located in regulatory or promoter regions of genes, and their methylation status is associated with gene suppression [237]. Studies have shown that several genes are differentially methylated in NAFLD patients compared to healthy individuals [236]. Specifically, through next-generation sequence analysis of liver samples, it has been observed that the level of global DNA methylation tends to be significantly decreased in NAFLD [237]. In an array-based DNA methylation study, Ahrens et al. identified differences in methylation in genes encoding for enzymes involved in metabolism (PC, ACLY, PLCG1) and insulin-related signalling pathways (IGF1, IGFBP2 and PRKCE) [238]. A case-control study by Sookoeian et al. identified that promoter methylation of PGC1-α, which is a regulator of mitochondrial biogenesis and fatty oxidation, was increased and correlated with insulin resistance in patients with NAFLD [239].

Changes in DNA methylation have also been identified in NAFLD disease progression between mild and advanced status, and it has been suggested that they may be highly predictive of fibrosis [240]. Murphy et al. analysed the methylome profile in patients with NAFLD and found that, in advanced NAFLD, 52,830 CpG sites (11%) were hypomethylated and 16,417 (3%) were hypermethylated, indicating that the advanced stage is characterised by loss of gene regulation [241]. Similarly, more hypomethylated than hypermethylated genes were found in advanced NAFLD vs. mild stage [241]. Pathway analysis revealed that hypomethylated genes were those associated with advanced fibrosis and cirrhosis (CO*L1A1*, *COL1A2*, *COL4A1*, *COL4A2*, *LAMA4*, *LAMB1*, *CTGF* and *PDGFA*) as well as with proinflammatory immune response (*STAT1*, *TNFAIP8* and *CASP1*) [241].

Over the last decades, research has started focusing on less invasive approaches. Towards this direction, current efforts are made in investigating methylation alterations in the peripheral blood rather than in liver biopsy samples [242]. A GWAS DNA methylation study by Zhang et al. in peripheral leukocytes discovered that the methylation profile is different between NAFLD patients and healthy individuals [241]. This study identified *ACSL4*, *CPT1C*, *IRS4* and *IKBKG* as critical genes that were hypomethylated and were associated with hepatic steatosis. Patients with *ACSL4* hypomethylation demonstrated an increased risk of NASH adjusted for BMI and HOMA-IR (OR 8.56, 95% CI, 1.33–54.95, *p* = 0.024) [241]. Another study involving European participants by Ma et al. identified differences in DNA methylation at 22 CpG sites, which were annotated to 18 genes, including (*DHCR24*, *SLC43A1*, *CPT1A*, *SREBF1*, *SC4MOL* and *SLC9A3R1*) [243]. Enrichment analysis showed that positive regulation of cholesterol biosynthesis was the most significantly enriched biological process [243].

It is worth noting that DNA methylation does not only occur in the nuclear genome, but it can also be found in the mitochondrial DNA [231]. Pirola et al. compared liver biopsies from NAFLD patients with healthy individuals and found that the mitochondria-encoded NADH dehydrogenase 6 (*MT-ND6*) has been shown to be significantly hypermethylated in patients with NASH in comparison with those who had simple steatosis, followed by decreased mRNA and protein *MT-ND6* levels [244]. Additionally, it was found that the DNA methyltransferase was upregulated in NASH patients and that the degree of methylation was associated with NAFLD severity [244]. Further studies are needed to elucidate and understand the role of the mtDNA methylation patterns and their potential association with NAFLD [245]. The execution of a wide methylation analysis in mtDNA will provide further possibilities in the area of diagnostic biomarker development in NAFLD [245].

#### 4.2.2. Histone Modifications

Histone modifications are important epigenetic changes that include post-translational modification of histone proteins, such as acetylation, methylation, phosphorylation and ubiquitylation. These modifications can regulate transcriptional activity by altering the chromatin structure [246]. Lysine acetylation is the most widely studied modification and is catalysed by histone acetyltransferases (HATs), leading to looser chromatin that promotes gene transcription, whereas deacetylation is conducted by histone deacetylases (HDACs) [247]. Despite acetylation being a well-known epigenetic mechanism, the alteration in the acetylation profile in patients with NAFLD has regained more attention only recently [248]. The carbohydrate-responsive element-binding protein (ChREBP) is an example of NAFLD modulation through acetylation [247,248]. The p300 is a transcriptional coactivator and belongs to the HAT family, which acetylates ChREBP, in high glucose state [248]. This increases lipogenic genes expression, such as SREBP1c and PPRARγ, leading to hepatic lipid accumulation [248,249]. The evidence regarding the diagnostic ability of p300 is limited, as it has been studied more as a potential therapeutic marker rather than a diagnostic biomarker. Tannic acid and rutin have been experimentally found to inhibit p300 HAT activity, therefore, suppressing the progression of NAFLD in mice [248,250]. Sirtuins (SIRTs) belong to the HDAC family, which have been found to play a protective role against NAFLD through deacetylation [248]. SIRT1 is an important protein that participates in regulating liver metabolism and insulin sensitivity, and it has antihyperlipidemic and anti-inflammatory properties. SIRT1, as well as SIRT3, SIRT5 and SIRT6, have been shown to be downregulated in patients with NAFLD with subsequent upregulation of lipogenic genes, including SREBP-1, FASN and ACC [251]. Experimental in vivo studies have shown that SIRT1, as well as SIRT3 deficiency, results in lipid accumulation and hepatic steatosis through acetylation, causing an overexpression of lipogenic, inflammatory and fibrogenic genes [248,252,253,254].

### 4.3. Transcriptomics

Transcriptomics include the quantitative assessment of coding, as well as non-coding RNA (ncRNA), and provide information related to differential gene expression and gene regulatory mechanisms [207]. Since coding transcriptomics is considered a molecular bridge between the genetic information from DNA to protein profile, research efforts are ongoing with regard to their potential role as diagnostic biomarkers in NAFLD [134]. Additionally, non-coding RNAs produced by the affected liver could potentially be used as biomarkers for evaluating dynamic changes that may occur during the progression of the disease, serving as diagnostic or predictive markers [134].

#### 4.3.1. Coding RNAs

Alterations in gene expression determine NAFLD transcriptomic signatures, which are characterised by the up or downregulation of various genes linked to biological processes responsible for NAFLD development and progression [247]. Therefore, studies that aim to understand the molecular mechanisms in NAFLD development and progression via observing transcriptomic changes are emerging [255]. Identifying specific patterns in gene expression can be useful in enlightening the complexity behind NAFLD/NASH; it may also aid in identifying novel diagnostic biomarkers [255]. The implementation of bioinformatics analysis of publicly available gene expression omnibus (GEO) GEO datasets for differential gene expression has led to the proposal of gene signatures that link with NAFLD [247]. Wang et al. identified that the transcriptomic profile is different between healthy obese individuals and obese patients with steatosis and NASH, as over 500 differentially expressed genes (DEGs) were observed [256]. This study identified *PRKCA*, *EGFR*, *CDC42* and *VEGFA* as important upregulated genes across the NAFLD spectrum [256]. Enriched pathways included FCγ-R-mediated phagocytosis and focal adhesion, which is a crucial process in liver disease, as it progresses from steatosis to steatohepatitis [256]. A more comprehensive analysis by Huang et al. showed that NAFLD was characterised by an upregulation of *CD24*, *THBS2*, *COLI1A1*, *LUM* and *EPHA3* and by a downregulation of *PZP* [257]. *CD24* demonstrated a robust differential expression and was proposed as a potential future biomarker for NAFLD; however, its precise mechanism of action remains unknown [257]. Enriched pathways were found to be related to metabolic processes, including glycolysis/gluconeogenesis and metabolism of glycine, serine and threonine [257]. *THBS2* has recently been proposed by Kozumi et al. as a potential biomarker for advanced fibrosis and NASH, as it has been found to be significantly overexpressed in patients with fibrosis, and its circulating protein product TSP-2 (thrombospondin 2) was shown to be an independent predictor of NASH [258]. *THBS2* was found to demonstrate the highest diagnostic accuracy for NASH with an AUROC of 0.96 in comparison with other genes and clinical scores, such as NFS (AUROC 0.88) and FIB-4 (AUROC 0.84) [258]. Intrahepatic *THBS2* and serum TSP-2 levels were found to be significantly associated with inflammation and hepatocellular ballooning [258]. Hepatic and circulating mRNA IL32 levels have also been found to be robustly upregulated in NAFLD and to be associated with the severity of the disease, independently from the presence of the *PNPLA3* variant [259]. Additionally, the combination of IL32 and ALT/AST as a predictive diagnostic model was found to improve accuracy by 14% in identifying patients with NAFLD in comparison with ALT/AST alone (AUROC 0.92 vs. 0.81, *p* < 0.001), suggesting the IL32 could serve as a non-invasive biomarker for the presence and severity assessment of NAFLD [259].

#### 4.3.2. Non-Coding RNAs (ncRNAs)

ncRNAs include the small RNAs, which are short RNAs with 19–23 nucleotides and the long-non-coding RNAs (lncRNAs), which are larger molecules—approximately 200 nt [260]. They regulate gene expression at a transcriptional and post-transcriptional level [260]. The snRNAs include the microRNAs (miRNAs), piwi-interacting RNAs (piRNAs) and the small nuclear RNAs (snRNAs).

Alternation in miRNAs expression and networks have been implicated in NAFLD and have been associated with hepatosteatosis and NASH [242]. Circulating miRNAs are emerging as potential promising non-invasive biomarkers for NAFLD state and progression [242]. The most widely investigated miRNA, as an established alteration in NAFLD, is the miR-122, which accounts for 70% of all miRNAs expressed in the liver [242,261,262]. A meta-analysis by Liu et al. showed that serum miR-122 levels were upregulated in patients with NAFLD vs. healthy controls and NASH vs. NAFLD patients and demonstrated moderate diagnostic accuracy for NAFLD (AUROC 0.69) and NASH (0.82) [262]. From a mechanistic perspective, miR-122 seems to be upregulated in NAFLD mice, leading to downregulation of *SIRT1*, whilst lipogenic genes, such as *SREBP1, ACC1, ApoA5, FASN* and *SCD1*, become upregulated [263]. On the other hand, a knockdown of miR-122 alleviated lipid deposition through the upregulation of *SIRT1*, indicating that miR-122 acts through regulating SIRT1 and may be a useful diagnostic biomarker and therapeutic target in NAFLD [263]. Serum miR-192 has also been proposed as a potential biomarker, as it has been found to be constantly upregulated in NASH, helping in distinguishing NAFLD and NASH severity (AUROC 0.68) [262]. miR-192-5p is shown to be upregulated in NAFLD and NASH patients and positively correlated with increased transaminases and hepatic steatosis. In vitro studies reported that exosomal-derived miR-192-5p induced macrophage activation in liver cells through the upregulation of IL-6 and TNF-a, indicating that may be important in regulating hepatic inflammation, playing a critical role in NAFLD progression [264]. Several other circulating miRNAs have been detected in patients affected by NAFLD [265]. As non-invasive diagnostic markers, circulating miRNAs seem promising in diagnosing NAFLD and also for predicting the progression to NASH [265]. Kim et al. identified eight upregulated miRNAs (miR-15b-3p, miR-21-5p, miR-29b-3p, miR-126-5p, miR-151a-3p, miR-183-5p, miR-192-5p and miR-4449), a combination of which provided high diagnostic accuracy for NASH, with 92% sensitivity, 92% specificity and AUROC of 0.92 [265]. However, further studies are needed to determine further their diagnostic accuracy in NAFLD [261].

In recent years, studies regarding the role of lncRNAs as potential biomarkers in NAFLD have been accumulating [236]. GWAS analysis by Sun et al. has identified 1735 dysregulated lncRNAs in patients with NAFLD [266]. In vivo and patient RNA-based sequencing studies suggest that several lncRNAs may play crucial roles in the pathogenesis and progression of NAFLD through their function in lipid and glucose metabolism [267]. LncRNA steroid receptor RNA activator (SRA) is enriched in liver tissue and has been found to be upregulated in NAFLD [267]. Mechanistically, in vivo studies have shown that SRA acts through its target gene *FOXO1* and suppresses *AGTL1* activity, thus reducing FFA oxidation in hepatocytes and promoting hepatic steatosis [268,269]. UC372 is a highly conserved lncRNA in mouse, rat and human genome, which has been found to be upregulated in NAFLD patients [270]. UC372 seems to promote lipogenesis through driving lipid accumulation by suppressing miRNAs that regulate gene expression related to lipid synthesis and uptake [270]. LncRNA-H19, one of the foremost identified lncRNAs, has been found to be upregulated in patients with NAFLD, and exogenous overexpression leads to lipid accumulation in mice hepatocytes by upregulating genes such as *APOC3* and *SREBP* involved in lipid synthesis and storage [269,271]. Several other lncRNAs have been identified to be overexpressed in NAFLD (lncLSTR, MEG3, MRAK052686, lncHR1) and NASH (HOTAIR, RUNX1, POA4-AS, NEAT1) and may be considered as diagnostic panel [269]. Evidence regarding the diagnostic accuracy of lncRNAs in NAFLD is lacking, suggesting the need for further studies to investigate their role as diagnostic biomarkers [269].

### 4.4. Proteomics

Proteomics refers to the global analysis of the proteome, which includes the technological applications for the identification and quantification of proteins that are present in a cell, tissue or organism [272]. Since the proteome is downstream and closer to phenotype, in comparison with the genome or transcriptome, proteomic analyses are preferred in search of biomarkers for NAFLD diagnosis and progression [272]. However, proteomics studies are still limited due to the limitations of current technologies in the detection and accurate quantification of candidate proteins. In addition, the proteome is more complex and dynamic than the genome or transcriptome due to several post-translational modifications and splicing [247]. Nevertheless, the advances in proteomics technologies and applications and the use of mass spectrometry (MS), as well as protein microarrays, have enabled research progression in the identification of some proteins that are differentially expressed between NAFLD and non-NAFLD individuals, which may serve as candidate proteomic biomarkers [273].

A systematic review of proteomic studies using MS discovered 251 candidate biomarkers, of which 33 were validated by standard protein analysis methods [273]. Apolipoprotein A1, apolipoprotein B, lumican, albumin, actin and α-1-acid-glycoprotein 1 were the proteins that were found to be overexpressed in both liver and serum samples, suggesting that these proteins may be more promising candidates than others [273]. Another study that also used MS to compare the proteome between healthy obese individuals and obese patients with NAFLD identified and quantified 3379 proteins, of which 132 were overexpressed, and 84 were downregulated in NAFLD liver tissues [274]. Bioinformatics analysis showed that the upregulated proteins were located in the extracellular region, as well as the nucleus, and were found to affect extracellular matrix organisation and cell adhesion, and were involved in PPAR signalling and insulin growth factor binding [274]. On the other hand, proteins that were downregulated were located mostly in the mitochondria and were related to oxidative phosphorylation processes [274]. A study by Younossi et al., who analysed the phosphoproteome of the liver, as well as the serum proteome in NAFLD patients with or without NASH, identified 74 differentially expressed proteins (DEPs), which mostly belong to three phosphorylated signalling pathways: the phosphoinositide 3-kinase PI3K/AKT, the epidermal growth factor receptor (EGF/EGFR) and the apoptosis signal-regulating kinase 1 (ASK1)-MAPK signalling pathway [275]. Amongst the differentially expressed phosphoproteins, Tyk2 and ALDH were found to be independent predictors for NASH, whereas the combined proteome profiling of liver and serum identified that hepatic ASK1 S38 and p38 MAPK, as well as serum α2M and coagulation factor V, were associated with collagen deposition in patients with NASH, indicating an association with liver fibrosis [275].

Several studies have proposed protein signatures that were found to be linked with NAFLD and may aid in the discovery of non-invasive biomarkers [276,277,278]. For instance, DDPE, ANPEP, PIGR, TGFBI and APOE have been found to be associated with NAFLD and liver cirrhosis, with the former three proteins having been validated in an in vivo NAFLD model [276]. Additionally, another six protein signatures (SELE, IGFBP7, IGFBP5, IL-1R2, NAGK and Decorin) have been shown to differentiate NASH patients with F3/4 from F1/2 stage with high diagnostic accuracy (AUROCs 0.93-1) [277]. Similarly, Luo et al. proposed a proteomics-based classifier, consisting of 12 serum proteins (LTBP4, IGF-1, VCAM1, IL1SRI, IL18Bpa, TSP2, collectin kidney 1, SHBG, TCCR, LIFsR, FBLN3 and PLXB2), which were able to discriminate between patients with early and advanced fibrosis (AUROC = 0.83) [278]. The combination of a proteomic signature along with genomic and phenotypic data has the potential to increase the predictive accuracy for liver steatosis [277,279]. Using a multiplexed proteomic assay, Wood et al. identified eight proteins that were associated with steatosis in a multivariable analysis (ACY1, SHBG, CTSZ, MET, GSN, LGALS3BP, CHL1 and SERPINC1). Diagnostic accuracy improved by 8.7% with the addition of the PBPLA3 rs738409 genome variant and phenotypic variables (AUROC 0.935 for the multi-component classifier vs. AUROC 0.86 for the proteomic component alone) [279].

Proteomics-based research into the discovery of candidate biomarkers in NAFLD has been limited so far due to methodological and technical challenges [247]. However, with the use of next-generation microarrays, which have the advantage of being low cost, and high-throughput biomarker analyses, as well as with the implementation of nanotechnology and the developments in bioinformatics software acceleration, the discovery of NAFLD protein biomarkers is undoubtedly expected in the future [280].

### 4.5. Metabolomics

Metabolomics refers to a comprehensive study of the metabolome present in cells, tissues and biological fluids of an organism [281]. It includes small molecules and metabolic products, such as amino acids, fatty acids and carbohydrates [247]. Given their dynamic nature, metabolomics provide only a “snapshot” of the metabolic profile under certain conditions at a specific time [281]. With the use of high-throughput analytical technologies, such as nuclear magnetic resonance spectroscopy and mass spectrometry, there has been a rise in metabolomics studies in the discovery of biomarkers in NAFLD and other liver diseases [281].

#### 4.5.1. Lipidomics

Hepatic steatosis is characterised by a disruption in the balance between TG production and accumulation in the liver; therefore, lipidomics is recognised as a major subfield of metabolomics, and it refers to the comprehensive investigation of cellular lipids [282]. Lipidomics studies have offered a considerable benefit in improving the understanding of metabolic pathways that contribute to NAFLD and NASH [282].

The primary structural component of complex lipids is fatty acids, which are introduced through diet (15%), released during lipolysis by the adipose tissue (60%) or synthesised in the liver through de novo lipogenesis (25%) [282,283]. Studies have shown that patients with NAFLD demonstrate the altered fatty acid composition of lipids with an increase in total saturated fatty acids (SFA) [283,284]. Additionally, it has been shown that SFAs are associated with the disease severity and NASH, as their increase can lead to dysregulation of the mitochondrial metabolism, promotion of ROS accumulation, induction of ER stress and activation of proinflammatory/profibrotic processes in the liver. Although less lipotoxic, the monounsaturated fatty acids (MUFAs), and especially oleic (18:1 n9) and palmitoleic acids (16:1 n7), are also involved in NAFLD pathogenesis, as they have been found to be elevated both in NAFLD patients and mouse NAFLD models [284,285,286].

On the other hand, polyunsaturated fatty acids (PUFAs) were found to be decreased in NAFLD patients [284,285]. PUFAs include two classes: the ω3 (such as the eicosapentaenoic acid (EPA, 20:5n-3) and docosahexaenoic acid (DHA, 22:6n-3) and ω6 FA (such as dihomo-γ-linolenic acid (20:3n-6) and arachidonic acid (20:4n-6) [282]. Lipidomics studies have reported a significant association between the decrease in hepatic PUFAs and the severity of NAFLD [286,287]. The ω6/ω3 ratio has also been found to play an important role in NAFLD development and progression, and studies have shown that an increased ω6/ω3 ratio has been observed in liver biopsies from patients with NASH vs. controls [285,287,288].

Glycerophospholipids are important components of cellular membrane, and phosphatidylcholine (PC), which is derived from the conversion of phosphatidylethanolamine (PE), is a vital cellular membrane lipid [284]. PC has been found to be decreased in patients with NAFLD, and lipidomics studies have shown that the PC/PE ratio in both liver and erythrocytes is decreased in NAFLD patients, and it has been proposed as a potential biomarker in NAFLD [289]. Sphingolipids are a subgroup of phospholipids that have also been found to be dysregulated in patients with NAFLD, and their dysregulation is potentially associated with disease progression [284]. Ceramides are a type of sphingolipids that have been found to be linked with insulin resistance and be positively associated with NAFLD severity and progression to fibrosis through ER stress, ROS production and proinflammatory cytokine secretion [290]. Recently, studies have shown a potential role of circulating amino acids, as well as bile acids, as non-invasive biomarkers for NAFLD severity. Gaggini et al. reported an increase in branched-chain amino acids (alanine, glutamate, isoleucine and valine) in patients with NAFLD, and they were associated with hepatic ballooning and inflammation [291]. Additionally, this study found that the glutamate/(serine + glycine) index was able to differentiate fibrosis F0–2 from F3–4 [291]. Bile acids have been investigated to a lesser extent, and most studies have shown increased levels of circulatory bile acids in patients with NASH and steatosis and a positive association with insulin resistance [282].

Since disturbances in lipid metabolism are important in the pathogenesis of NAFLD, lipidomics studies are emerging to identify lipidomic signatures that could serve as non-invasive biomarkers in the diagnosis of NAFLD and predict disease progression [292,293,294,295]. Hu et al. compared NAFLD with the healthy group and identified 15 serum metabolites, which demonstrated an AUROC above 0.9, indicating a high diagnostic value. Metabolite pathway enrichment analysis revealed a pathway signature that included phenylalanine, glycerophospholipid and ether lipid metabolism, fatty acid biosynthesis and tricarboxylic acid cycle [296]. Jung et al. investigated NAFLD patients who were separated into obese and non-obese cohorts [297]. This study identified five lipid metabolites (TAG 46:1, TAG 48:1, TAG 50:1, SM d32:0 and SM d38:0A) in the non-obese group, with high diagnostic accuracy for NAFLD vs. control and NAFLD vs. NASH, with AUROC of 0.92 and 0.81, respectively [297]. Additionally, for the obese group, seven lipid metabolites (DAG 34:1, DAG 40:7, DAG 40:8, TAG 46:1, TAG 48:1, TAG 50:2 and SM d36:0) were identified to be potential diagnostic biomarkers for NAFLD and NASH, with AUROCs of 0.97 and 0.81, respectively [297]. A large lipidomics study by Mayo et al., which used various mathematical models, proposed two triglyceride panels [292]. The former consisted of 11 triglycerides and could differentiate between NAFLD and healthy individuals with 94% sensitivity, 57% specificity and AUROC of 0.88. The latter metabolomic panel consisted of 20 triglycerides, which could differentiate between NAFLD and NASH patients with 70% sensitivity, 81% specificity and AUROC of 0.79 [292]. However, these algorithms performed poorly in a group of patients with T2DM (AUROC = 0.69) [247]. Masarone et al. used machine learning and mathematical models to create a metabolomic profile for the prediction of NAFLD presence and stage, including clinical and biochemical variables [298]. Several metabolites were identified, which showed progressively increased (isocitric acid, isoleucine) or decreased concentration (xanthine, glutathione, glycolic acid) in NAFLD towards NASH vs. controls [298]. Metabolic pathway profiles include dysregulation of amino acid metabolism, glycolysis and gluconeogenesis, TCA cycle, bioprotein metabolism and butanoate metabolism [298]. The metabolic profile of controls, NAFLD and NASH patients was significantly different, and these alternations were able to distinguish between the groups with high diagnostic accuracy (AUROC 0.99 for control vs. NAFD and NAFLD vs. NASH). Interestingly, butanoate metabolism has been recently identified as a top dysregulated metabolic pathway in NAFLD and a potential novel lipidome-related phenotype, which may also interact with microbial-derived metabolites for the pathogenesis of NAFLD [299].

#### 4.5.2. Glycomics

Glycomics refers to a new “omics” approach that has emerged and received research attention over the last twenty years [277]. Glycomics includes a comprehensive analysis of glycan structures that exist either as free glycans or are bound to proteins [247]. Glycans are sequences of carbohydrates that are added to proteins or lipids, resulting in changes in their structure and function. Protein glycosylation is a well-known post-translational modification catalysed by glycosyltransferases, causing a significant diversity in the glycome [300]. Alterations in protein glycosylation have been found to be associated with the pathogenesis of various diseases, such as inflammatory bowel disease, colorectal cancer, NAFLD, liver fibrosis and hepatocellular carcinoma [300]. Chen et al. have found glycan alterations between NAFLD and NASH patients and proposed the GlycoNASHTest, which is determined by the serum logarithmic ratio of two glycans: the core fucosylated galactosylated biantennary glycan (NGA2F) vs. the agalactosylated non-fucosylated biantennary glycan (NA2) [301]. The GlycoNASHTest was found to be able to identify NASH patients within the NAFLD group with an accuracy of 70% and with a sensitivity and specificity of 45% and 90%, respectively [301]. Similarly, in a large cross-sectional study involving 500 participants, Zhao et al. showed that IgG *N*-glycosylation was associated with NAFLD, with fucosylation demonstrating a positive correlation with the disease, whereas galactosylation showed a negative correlation after being adjusted for clinical and biochemical variables [302]. The GlycoFibro and GlycoCirrho tests also measure the presence of glycans, and the former has been found to be exponentially increased as the fibrosis stage progresses, whilst the latter has been shown to identify patients with cirrhosis who are at risk of developing hepatocellular carcinoma [300,303].

Fucosylation of other glycans, such as alpha-1 antitrypsin, haptoglobin, transferrin and ceruloplasmin, has been shown to be related to the progression of NAFLD to NASH, and higher concentrations have been observed during NAFLD progression to liver fibrosis [304,305]. Very few glycomics studies have been conducted so far, mainly due to the high complexity and cost of current technologies in quantifying glycans and other molecules [277]. Despite the observed changes in the glycome between NAFLD patients and healthy individuals, the current evidence is not sufficient to conclude whether the glycome alone offers diagnostic accuracy in identifying NAFLD or predicting NAFLD progression [247,277]. A recent comprehensive metabolomics study by Perakakis et al. aimed to identify metabolomic changes and to build predictive omics models for NALFD and NASH, including lipids, fatty acids and glycans [295]. This study observed that glycans alone were poor at discriminating between NAFLD/NASH and the healthy group (sensitivity 64%, specificity 81%, AUROC 0.77). This study showed that lipids alone (sensitivity 89%, specificity 94%, AUROC 0.87–0.95) or in combination with glycans and hormones had higher accuracy in diagnosing NASH vs. NAFLD vs. healthy individuals (sensitivity 84–87%, specificity 92–94%, AUROC 0.83–0.96) [295].

### 4.6. Metagenomics

Whilst genomics defines the whole genetic complement of an organism, metagenomics determines the genomic sequence of organisms inhabiting a common environment [306]. This approach has been applied mainly to microbial communities and is defined as the genomic analysis of the microbial DNA [306].

The gut microbiome interacts with the liver through the gut–liver axis, which is facilitated by the portal vein that enables the transport of products derived by the gut to the liver [307]. Emerging evidence supports the potential role of the microbiome and its derived metabolites in the pathogenesis of NAFLD [307]. A large metabolomic and metagenomic study by Caussy et al. found that among the 56 identified altered metabolites, 6 of them were gut derived and demonstrated shared gene effect with liver steatosis and fibrosis [308]. The top metabolite was identified to be 3-(4-hydroxyphenyl) lactate, which was consistently associated with advanced fibrosis and was significantly correlated with the overgrowth of several gut microbiome species from Firmicutes, Bacteroidetes and Proteobacteria phyla families [308]. Alterations in gut microbiota, known as dysbiosis, are known to be associated with liver diseases, including NAFLD and HCC [309]. The exact mechanism of how gut microbiota may contribute to the development and progression of NAFLD is unclear [310]. Clinical and pre-clinical studies suggest that dysbiosis may lead to overgrowth of pathogenic commensals, which can increase gastrointestinal permeability, which can lead to the production of microbial-derived metabolites, which can affect immunity and trigger tissue and systemic inflammation [309,310].

Recent metagenomics research indicates that some microbiota members may serve as biomarkers in the diagnosis and prognosis of NAFLD. Caussy et al. and Ponziani et al. found that microbial species belonging to Enterobacteriaceae and Streptococcus were more enriched in NAFLD-associated cirrhosis patients [311,312]. Loomba et al. proposed a metagenomic diagnostic model consisting of 37 species in combination with age, BMI and species’ diversity, which demonstrated high diagnostic accuracy (AUROC = 0.94) in the detection of advanced fibrosis [313]. Interestingly, metagenomics studies have shown that there seems to be a universal shift in the microbiota towards gram-negative bacteria in the NAFLD patients with advanced fibrosis [309]. In addition to the microbiota, alterations studies have also investigated gut-derived metabolomic signatures in the diagnosis and progression of NAFLD [309,310]. Gut-derived metabolites include lipopolysaccharides (LPS), short-chain fatty acids (SCFAs) such as butyrate and acetate, products of bile acid metabolism, ethanol and 3-(4-hydroxyphenyl) lactate, a product of aromatic amino acid metabolism [309]. Undoubtedly, a further increase in metagenomics research in NAFLD is expected, as a growing body of evidence suggests that gut microbiota is distorted by environmental factors and may drive NAFLD development. Therefore, it may be important in the identification of future non-invasive biomarkers for the diagnosis and prediction of NAFLD progression [309].

## 5. Current Challenges and Future Directions

Liver biopsy is still considered the gold standard investigation for NAFLD, as it provides an evaluation of liver steatosis, hepatocellular injury and inflammation [314,315]. Additionally, liver biopsy provides the advantage in that it can differentiate NASH from simple steatosis and other liver diseases, as well as assess the activity and degree of fibrosis [314,315]. However, liver biopsy exerts several diagnostic limitations, including sampling error and inter-observer variability [316]. Considering the inconsistency of histological features in NASH, the use of a small liver sample can lead to an inaccurate diagnosis. Moreover, the characterisation of the fibrosis stage can be biased by the variability in the assessment from different observers [316]. Lastly, liver biopsy is an invasive procedure, entailing post-procedural complications ranging from minor, such as pain (20%), to major, such as bleeding (0.3%), and even mortality (0.01%) [316]. Due to the invasive nature of the liver biopsy and its potential limitations, developing novel non-invasive diagnostic techniques is one of the most rapidly advancing fields during the last decades. Significant progress has been noted in the imaging diagnosis of liver fibrosis, and further development of these techniques is anticipated in the future. However, despite the promising outcomes of the new non-invasive imaging diagnostic methods, their use in clinical practice is currently limited. The high cost and the need for special equipment, as well as trained personnel, has restricted their availability, and their use is often limited to research purposes [164,185]. Additionally, detecting early fibrosis stages with accuracy appears to be a common limitation between several non-invasive techniques [160]. Nevertheless, further advancements in the current non-invasive imaging modalities and biochemical indices are needed and expected for accurate quantification of fatty liver changes and differentiation between NASH and simple steatosis, with the aim to be considered equal replacements and become established alternatives to liver biopsy.

In the era of non-invasive biomarker discovery, omics is a promising revolutionary approach, as it provides the benefit of a large-scale analysis of patients’ samples [317]. The advancement in omics technology has allowed the identification of promising novel biomarkers in a hypothesis-free approach [134]. In addition, machine-learning-based-methodologies are constantly evolving to discover multi-omics marker panels and explore their diagnostic accuracy [134]. Pioneering studies are now investigating the integration of multi-omics data with clinical characteristics to build multi-component classifiers for diagnosing NAFLD [317,318]. Zhou et al. developed the NASH ClinLipMet Score, including PNPLA3 genotype, metabolomics and lipidomics data, as well as clinical variables [318]. The NASH ClinLipMet score demonstrated a sensitivity of 86%, specificity of 71% and an AUROC of 0.87 for diagnosing NASH. The multi-component panel significantly improved the diagnosis of NASH by 11–20% when compared with clinical or omics data alone [318].

**Figure 3:**
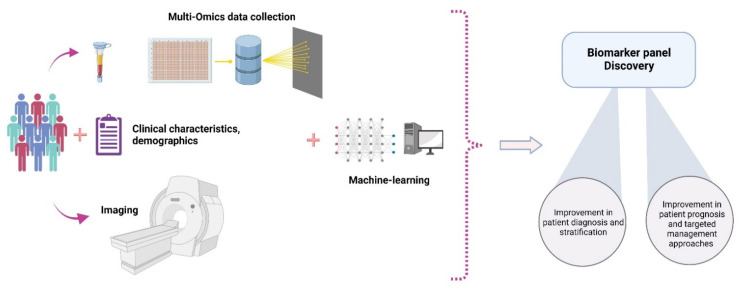
Future directions in the era of non-invasive diagnosis of NAFLD by integrating omics big data with clinical information and imaging (created with BioRender.com, accessed on 19 December 2021).

The ultimate purpose to replace the liver biopsy with accurate and highly specific non-invasive biomarkers remains [247]. Rigorous omics-based research that includes discovery, validation and replication steps is mandatory before omics biomarkers are ready to be translated into clinical practice [231]. Despite the remarkable advances in multi-omics technologies, diagnostic multi-omics models are yet to reach the desired level of performance to be incorporated into clinical practice due to existing challenges, such as high cost and reduced availability [247]. Moreover, omics-based studies face challenges with regard to the acquisition of big data and the technologies required for data storage and analysis [319]. Undoubtedly, with the advancement in omics sequencing technologies, computational biology software and artificial intelligence, larger-scale omics studies will be conducted in the future to aid the discovery of diagnostic biomarkers in NAFLD. In the era of personalised non-invasive biomarker discovery, the integration of omics data, clinical characteristics and imaging information will facilitate the diagnosis, stratification and prognosis of NAFLD patients (Figure 3).

## 6. Conclusions

In conclusion, this review highlights that there have been significant developments in non-invasive assessment for NAFLD and NASH. Thus, several blood-based biochemical markers have been identified, and their combination (NFS, FIB-4 score) has improved diagnostic accuracy and stratification within the NAFLD spectrum. Additionally, new imaging techniques have emerged, offering high diagnostic accuracy for steatosis and fibrosis, with MRI-PDFF being a promising modality. However, cost and availability remain a challenge for their wide application. Omics have offered a major evolution in the era of non-invasive biomarker development and have significantly improved the understanding of molecular and metabolomic pathophysiology in NAFLD. With the advancement in machine learning and artificial intelligence, several multi-omics and multi-component diagnostic scores have been developed. However, the implementation of omics-derived biomarkers still requires further investigation prior to their implementation in the diagnostic clinical practice.

## Figures and Tables

**Table 1:** Non-invasive biochemical diagnostic models for predicting the presence of NAFLD, NASH and advanced fibrosis. The table includes proprietary and non-proprietary tests.

Diagnostic Tool	AUROC	SS (%)	SP (%)	PPV (%)	NPV (%)	Refs
Non-invasive biochemical biomarkers predicting the presence of NAFLD						
SteatoTest	0.79	91	70	63	93	[36,37]
Fatty Liver Index (FLI)	0.85	87	86	--	--	[38]
NAFLD Liver Fat Score	0.88	86	71	--	--	[41]
Non-invasive biochemical biomarkers and models predicting the presence of NASH						
Cytokeratin-18 (CK-18) or KRT18	0.82	78	87	--	--	[44,45]
Soluble Fas and Fas Ligand	0.71	88	89	--	--	[50]
oxNASH score	0.74	84	63	62	74	[56]
HAIR score (hypertension, ALT and insulin resistance)	0.90	80	89	80	89	[64]
Gholam score	0.90	--	--	--	--	[65]
Palekar score	0.76	74	66	--	--	[66]
NAFIC score (Ferritin, insulin and collagen)	0.85	94	48	--	--	[68]
NashTest	0.79	33	94	--	--	[70]
NIS4 score	0.80	87	51	79	78	[71]
Non-invasive biochemical diagnostic models predicting the presence of advanced fibrosis						
FibroTest™	0.86	77	98	73	90	[99]
European Liver Fibrosis panel (ELF)	0.87	90	41	35	92	[101]
Hepascore	0.82	67	76	63	79	[105]
FibroSpect II (Prometheus Corp)	0.83	82	66	74	76	[108]
NAFLD fibrosis score (NFS)	0.88	82	98	90	93	[109]
Fibrosis 4 (Fib-4) score	0.76	70	97	80	90	[114]
AST/ALT ratio	0.66	53	100	100	81	[118]
AST/platelet ratio index (APRI)	0.87	61	64	40	81	[124]
BAAT (BMI, age, ALT and triglycerides) score	0.84	100	47	45	100	[129]
Nippon score	0.78	84	92	--	--	[130]
BARD (BMI, AST/ALT, diabetes) score	0.81	89	89	43	96	[130]
ADAPT (age, diabetes, PRO-C3 and platelet count) score	0.86	91	73	48	97	[131]

ALT: Alanine aminotransferase, AST: Aspartate aminotransferase, AUROC: Area Under the Receiver Operating Characteristic curve, BMI: Body mass index, HAIR: Hypertension, NAFLD: Non-alcoholic fatty liver disease, NASH: Non-alcoholic steatohepatitis, NAFIC: Ferritin, insulin and collagen, PRO-C3: N-terminal pro-peptide of type III collagen, NPV: Negative Predictive Value, PPV: Positive Predictive Value, SS: Sensitivity, SP: Specificity.

## Data Availability

All data presented in this study are included in the paper.
